# ACCEPT 3.0: Recalibrating ACute COPD Exacerbation Prediction Tool (ACCEPT) for global use

**DOI:** 10.1186/s41512-026-00242-x

**Published:** 2026-10-08

**Authors:** Laura Huey Mien Lim, Jeenat Mehareen, Amin Adibi, Christer Janson, Pierre-Régis Burgel, Tae Yoon Lee, Hana Müllerová, Della Varghese, Jonathan Marshall, Surya P. Bhatt, Don Sin, Wenjia Chen, Mohsen Sadatsafavi

**Affiliations:** 1 Respiratory Evaluation Sciences Program, Faculty of Pharmaceutical Sciences, University of British Columbia, 2405 Wesbrook Mall, Vancouver, BC V6T1Z3 Canada; 2 Saw Swee Hock School of Public Health, National University of Singapore, Singapore, Singapore; 3 BHF Cardiovascular Epidemiology Unit, Department of Public Health and Primary Care, University of Cambridge, Cambridge, UK; 4 Department of Medical Sciences, Respiratory, Allergy and Sleep Research, Uppsala University, Uppsala, Sweden; 5 Department of Respiratory Medicine, Cochin Hospital, Assistance Publique-Hôpitaux de Paris, Paris, France; 6 Université Paris Cité, Institut Cochin, Inserm U1016, Paris, France; 7 Quantitative Sciences Unit, Stanford University School of Medicine, Palo Alto, CA 94304 USA; 8 Respiratory Evidence Strategy, BioPharmaceuticals Medical, AstraZeneca, Cambridge, UK; 9 Respiratory Evidence Strategy, BioPharmaceuticals Medical, AstraZeneca, Gaithersburg USA; 10 Global Medical Affairs - Respiratory, BioPharmaceuticals Medical, AstraZeneca, Cambridge, UK; 11 Center for Lung Analytics and Imaging Research, Division of Pulmonary, Allergy and Critical Care Medicine, University of Alabama at Birmingham, University of Alabama at Birmingham, Birmingham, AL USA; 12 UBC Centre for Heart Lung Innovation (HLI), St. Paul’s Hospital, Vancouver, BC Canada; 13 Division of Respiratory Medicine, University of British Columbia, Vancouver, BC Canada

**Keywords:** COPD, Exacerbation, Risk prediction, Country variation

## Abstract

**Background:**

The ACute COPD Exacerbation Prediction Tool (ACCEPT) is an algorithm for predicting 12-month risk of chronic obstructive pulmonary disease (COPD) exacerbations. To make it suitable for international use, we externally validated and recalibrated ACCEPT 2.0 to incorporate country-specific background exacerbation risk, using multi-country data from NOVELTY (a NOVEL observational longiTudinal studY).

**Methods:**

Our study cohort included patients aged $\:\ge\:$40 years with physician-diagnosed COPD and $\:\ge\:$30 days of follow-up. We predicted the occurrence of any moderate-to-severe (primary endpoint) and severe (secondary) exacerbations at 12 months, using country-level random effects to account for between-country differences in background exacerbation risks unexplained by predictor effects. We assessed model performance via discrimination, calibration, and net benefit.

**Results:**

The analysis sample comprised 5,181 patients (59.4% male, mean age at baseline 66.5 years) from 18 countries, with a mean follow-up of 357 days. 12-month risks ranged between 0.11 and 0.46 (mean = 0.26) for moderate-to-severe and 0.03–0.21 (mean = 0.08) for severe exacerbations. Without country-level adjustment, ACCEPT 2.0’s area under the receiver operating characteristic curve (AUROC) was 0.73 (95% CI 0.72–0.75) and 0.76 (95% CI 0.73–0.78) for moderate-to-severe and severe exacerbations, respectively. However, ACCEPT 2.0 consistently overpredicted risks (observed-to-expected [O/E] risk ratio = 0.57 [95% CI 0.54–0.60] and 0.51 [95% CI 0.46–0.56]). After country-level recalibration, ACCEPT 3.0 showed similar AUROCs (0.75 and 0.76) but substantially improved calibration (O/E ratios: 0.99 [95% CI 0.94–1.05] and 0.99 [95% CI 0.90–1.09]). The net benefit of ACCEPT 3.0 exceeded ACCEPT 2.0 across treatment thresholds of 5%-20%.

**Conclusion:**

Recalibrating ACCEPT 2.0 with country-level effects significantly improved calibration of COPD exacerbation risks. The resulting ACCEPT 3.0 is expected to provide higher clinical utility across a range of treatment thresholds.

**Supplementary Information:**

The online version contains supplementary material available at https://doi.org/10.1186/s41512-026-00242-x.

## Introduction

Globally, chronic obstructive pulmonary disease (COPD) is a leading cause of mortality and health impairment [1]. Acute exacerbations of COPD (AECOPDs) or “flare-ups”, which refer to episodes of worsening symptoms, are a major cause of morbidity and mortality. Consequently, their prevention is a primary goal in the pharmacological management of COPD, as stipulated in the Global Initiative for Chronic Obstructive Lung Disease (GOLD) guidelines [2].

Owing to significant heterogeneity in biological mechanisms and clinical phenotypes, the importance of a targeted approach to COPD treatment is increasingly recognised [3]. Clinical prediction models support precision care by guiding risk-stratified treatment, enabling targeted interventions. While there are several existing AECOPD risk prediction models, most are not implementable due to lack of demonstrated external validity or high risk of bias stemming from the use of unsound statistical methods in model development and the choice of performance measures [4].

One of the more recent prediction algorithms is the ACute COPD Exacerbation Prediction Tool (ACCEPT) [5], which was built using pooled data from three randomised clinical trials and recalibrated in the ECLIPSE (Evaluation of COPD Longitudinally to Identify Predictive Surrogate Endpoints) cohort [6]. It combines AECOPD history – often considered as the best predictor of future risk [7] – with several other predictors that are routinely available at point of care (age, gender, smoking status, body mass index [BMI], lung function, domiciliary oxygen therapy, symptom burden, and current medication use) to predict 12-month risk of moderate-to-severe AECOPDs [5]. Due to specific attempts at validating ACCEPT and its demonstrated clinical utility [6, 8], it has been regarded as one of the few reliable tools for managing risk prediction for AECOPD [9].

The transportability of prediction models from one setting to another is an important challenge affecting the use of risk stratification algorithms [10], with recent evidence indicating that models are unlikely to be transportable especially if the outcome prevalence is significantly different across jurisdictions [11, 12]. In a large-scale validation study, Gulati et al. showed that prediction models for risk of cardiovascular diseases (CVDs) failed to generalise to new settings or populations, leading to substantial risk of harm, manifested as the net benefit of using the model being below that of not using it [12, 13]. This risk of harm was reduced when the models were adjusted for background CVD risk in the new population. Similarly, for ACCEPT, it has been shown that the performance of the model can be significantly improved if average AECOPD risk in the target population is taken into account [6]. Accounting for variability in outcome risk may be even more crucial for airway diseases, where exacerbations are defined in part by a subjective, symptom-based criteria and are thus often dependent on local practice patterns and cultural norms.

Country-level heterogeneity in AECOPD risk has been well-documented. Based on evidence from international randomised trials, there is a two- to three-fold difference in mean exacerbation rates across jurisdictions [13]. Evidence suggests that system-level effects beyond patient and disease characteristics are potentially at play [14, 15], including factors such as quality of care, local clinical practices (e.g., the implicit threshold for AECOPD severity that would invoke hospital admission), medication availability and costs, and air quality. Relying solely on salient predictors might not be sufficient to account for such variability. This necessitates a customised approach to risk profiling that accounts for systemic differences in AECOPD risk across populations.

Compared to developing a new model for each new target population, updating an existing model using external site- or country-level datasets allows well-captured predictive information from the original model to be preserved [10]. As such, we followed best practice standards [16] in model revision to create ACCEPT 3.0, a model that retains the core predictive function of ACCEPT 2.0 (the latest version of the model [6]) but enables the incorporation of background AECOPD risk in new populations. Specifically, we set out to validate and revise ACCEPT 2.0 to generate a new version of ACCEPT that is suitable for international use. We leveraged data from NOVELTY (a NOVEL observational longiTudinal studY), a contemporaneous, multi-country, observational study comprising ~ 12,000 asthma and COPD patients under routine primary and secondary care [17]. With pronounced emphasis on pragmatism, especially in the way airway diseases are diagnosed and classified, NOVELTY is considered the largest real-life study dedicated to asthma and COPD [18].

## Methods

Ethics approval for this study was obtained from the University of British Columbia’s Human Ethics Board (H11–00786). The NOVELTY study received approval from the institutional review boards of each participating institution, and written informed consent was obtained from all patient subjects [17]. This study was conducted in accordance with the Declaration of Helsinki, and the Transparent Reporting of a multivariable prediction model for Individual Prognosis Or Diagnosis (TRIPOD + AI) statement was used for reporting this study [19].

### Data source: NOVELTY

In this study, we used data from NOVELTY to perform external validation and recalibration of ACCEPT (underlying data and framework summarised in Additional file 1 Appendix 1). The NOVELTY study was a multi-national prospective cohort study with the primary aim of identifying new phenotypes and endotypes related to differential outcomes in asthma and COPD patients. Details of the study have been described elsewhere [17].

#### Patient eligibility

NOVELTY included patients aged $\:\ge\:$12 years who had a diagnosis or suspected diagnosis of asthma, COPD, or both, according to the discretion of the treating physician. Of note, the lack of spirometry diagnostic criteria was intentional, to reflect community practice. Other eligibility criteria included no participation in any interventional respiratory clinical trial during the pre-enrolment 12-month period and high likelihood of completing three years of follow-up. Patients were prospectively recruited between September 2015 and March 2017 from primary and specialist clinical practices across 18 countries. The baseline visit date was the date of consent for patients, unless they were enrolled during an exacerbation episode, in which case it was six weeks after the consent date.

All patients received standard medical care as determined by their physician. No experimental intervention was assigned as part of the NOVELTY study, and those who entered an interventional trial during the study were withdrawn. All recruited patients had yearly in-person visits and phone-based visits every three months and were followed up over three years. Data collected included clinical assessments, spirometry and biospecimens (captured yearly in electronic case report forms, coupled with data collected in electronic medical records for some countries) as well as patient-reported outcomes and healthcare utilisation (collected quarterly via a web interface or telephone). Data collection was standardised across study sites using electronic case report forms.

### Study design

**Fig. 1:**
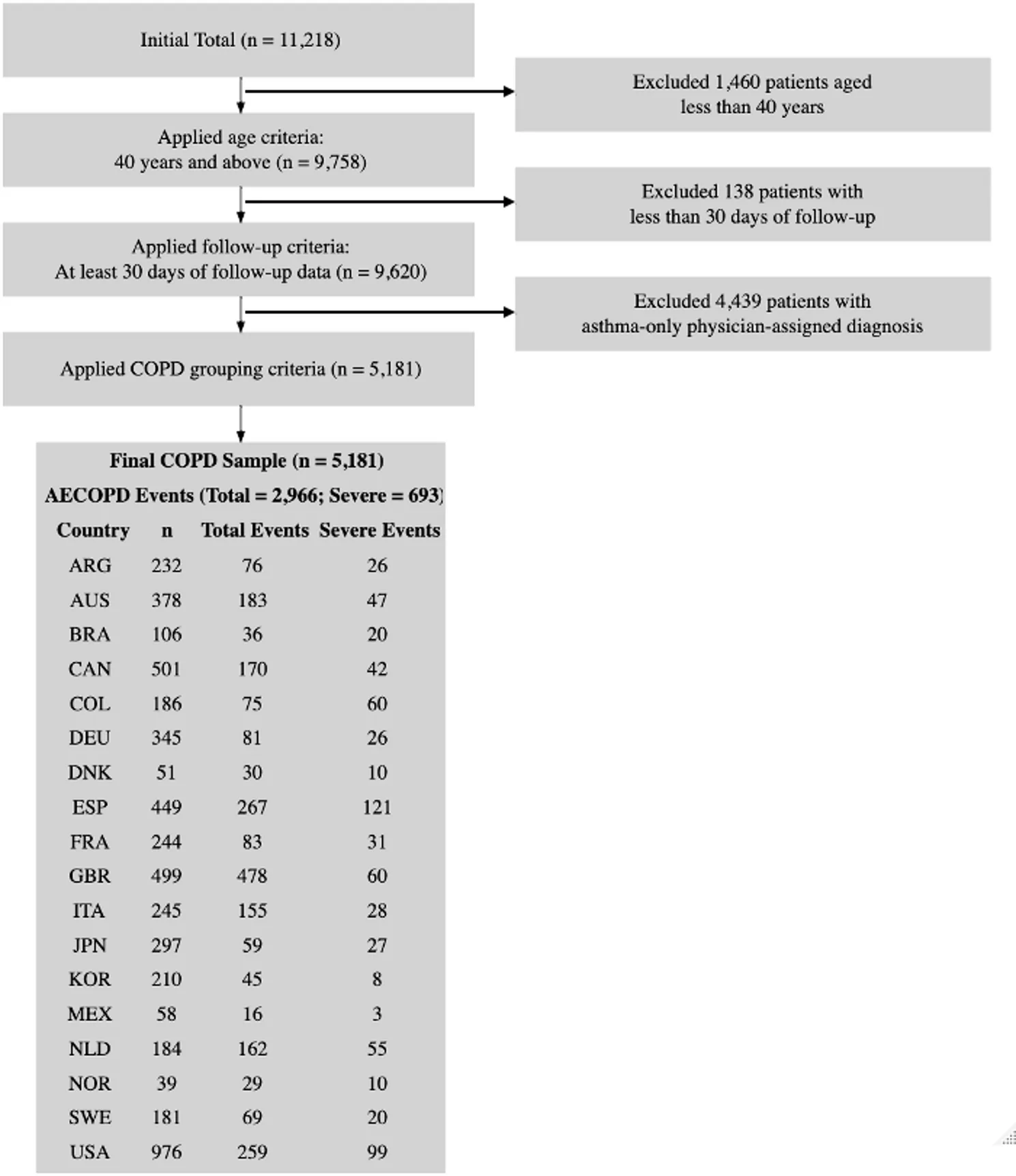
Displays the flow diagram of patient selection from NOVELTY. From the initial total of 11,218 patients, we excluded 1,460 patients aged < 40 years at baseline; 138 patients with < 30 days of follow-up; and 4,439 patients whose primary respiratory diagnosis at baseline was not COPD (i.e., “Asthma”). No patients were excluded due to unknown baseline visit date or end-of-follow-up date. The final sample consisted of 5,181 patients

We included patients aged $\:\ge\:$40 years with a physician diagnosis or suspected diagnosis of COPD and known dates of baseline visit (enrolment into NOVELTY) and end of follow-up. We defined the *index date* for each patient as their baseline visit date. Patients were excluded from the analysis if their primary baseline respiratory diagnosis i.e., the condition leading to most of their respiratory symptoms at baseline, was not COPD, or if they had <30 days follow-up. Figure 1 presents a flow diagram of patient selection. We followed each patient from their index date up to the earliest of these events: the occurrence of a moderate-to-severe AECOPD, death, loss to follow-up, or at month 12 of follow-up (time horizon of ACCEPT prediction).

### Variables

The primary outcome of interest was the occurrence of any (≥ 1) moderate-to-severe AECOPD over 12 months. The secondary outcome was the occurrence of any (≥ 1) severe AECOPD over 12 months. Exacerbations during follow-up were defined in accordance with European Respiratory Society/American Thoracic Society definition, i.e., episodes of worsening respiratory symptoms that required treatment with systemic steroids, antibiotics, or both [20, 21]. Exacerbations that resulted in an emergency department visit or hospitalisation were considered severe; otherwise, they were classified as moderate. All recorded episodes of exacerbation had known year (since baseline visit) of occurrence and therefore no episode was excluded from the analysis.

The predictors of AECOPD included baseline age, sex, smoking status (current smoker or otherwise), post-bronchodilator forced expiratory volume in one second (FEV_1_, % predicted), BMI, pre-index 12-month number of moderate and severe exacerbations, baseline COPD inhaled treatments (defined as any use of inhaled corticosteroids [ICS], long-acting beta agonists [LABAs] and long-acting muscarinic receptor antagonists [LAMAs], coded as three separate indicator variables). Exacerbation history was assessed initially by physicians as events “beyond the patient’s usual day-to-day variance” based on consensus recommendations [22]. Exacerbations were recorded at recruitment and on follow-up visits to the NOVELTY clinical site on an annual basis. If two AECOPDs occurred within 14 days of each other, they were considered one event. Although ACCEPT 2.0 used the St. George Respiratory Questionnaire (SGRQ) total score as a predictor, it also allowed the use of simpler tools such as the modified Medical Research Council (mMRC) dyspnoea score, which are used more commonly in clinical practice. We therefore used mMRC in this validation study. In ACCEPT 2.0, due to the limited availability of data in the development samples, concomitant use of statins had been taken as a surrogate for CVD. In NOVELTY, CVD history was collected at baseline through standardised questionnaires, and this was included as a predictor.

### Statistical analysis

All analyses were performed using R version 4.5.1. Descriptive statistics were derived for baseline variables. We reported the mean and standard deviation (SD) for continuous variables and the counts and proportions for categorical variables. Missing values in baseline variables were imputed using a robust, non-parametric machine learning-based algorithm *missRanger *[23], which performs univariate iterative imputation (allowing for a maximum of 10 iterations by default) and applies predictive mean matching to avoid outliers and recover the natural data variability [24]. To assess the plausibility of the missing-at-random (MAR) assumption underlying this approach, we followed the approach used by Clark and Altman [25] and Vergouwe et al. [26], and found that missingness was strongly patterned by study site (variation explained by country: 11.0%-26.5% [Nagelkerke R^2^]) and substantially explained by observed patient characteristics (missingness of domiciliary oxygen therapy [log-rank *P* < 0.001] and mMRC [*P* = 0.003] were associated with time to first moderate-to-severe exacerbation); as such, we consider MAR a plausible assumption for this data. [25, 26]. Incomplete month or date information of exacerbation events, which was found in 977 out of 5,958 observations (16.4%, of which 13.3% lacked day information while 3.1% lacked both month and day information), was imputed with the most recent time, i.e., last day of the month and/or last month of the year. For robustness checks, we re-generated predicted risks by re-dating these episodes to the earliest possible date, and the effect seen was small (< 1% change in outcome across all events).

#### Model recalibration

To adjust the distribution of predicted risks, we recalibrated ACCEPT 2.0 by applying country-specific random intercepts and a fixed correction slope to predicted risks. We followed the methodology explained by McLernon et al. for recalibrating prediction models for survival outcomes [16]. We fitted a mixed-effects Cox model with the observed survival time as the dependent variable, and the transformed 12-month risk from ACCEPT 2.0 (via the complementary-log-log link) as the independent variable. Country was included as a random-effect (frailty) term to account for country-level variation in outcome risk. The random intercept for each country acts as a correction factor that adjusts for systematic calibration error, while the coefficient of the transformed predicted risks is a single slope-correction factor (identical across countries). This model structure was selected due to its parsimony and after an exploratory analysis considering alternative fixed- and random-effect models.

For the secondary outcome (severe AECOPD risk), we used the same set of country-specific intercepts but a re-estimated slope, as an exploratory analysis showed that re-estimating country effects separately for this outcome did not provide any further improvement in model performance. Appendix Figure A1 in Additional file 1 summarises the steps of model recalibration for the two outcomes.

To further extend the use of this model to any setting where the background AECOPD risk is known (not just the 18 countries included in NOVELTY), we used the observed relationship between AECOPD risk and the estimated random intercept for each country to develop a function for mapping a given AECOPD risk to an intercept adjustment term.

#### Model validation

We followed the best practice recommendations on time-to-event model validation [16]. We evaluated the performance of ACCEPT 2.0 and its recalibrated form (ACCEPT 3.0) in NOVELTY in terms of fixed-timepoint discrimination and calibration. All assessments were based on 12-month risks.

We assessed model discrimination by the area under the receiver operating characteristic curve (AUROC), which measures the ability of the model to discriminate between high- and low-risk individuals. The inverse probability of censoring weighting method developed by Uno et al. was used for estimating AUROC [27]. Model calibration measures the degree to which the expected risks agree with the observed risks. We measured “calibration-in-the-large” as the ratio of the observed survival proportion (i.e., the Kaplan-Meier estimate of outcome occurrence at 12 months) and the mean predicted risks [16]. An observed-to-expected (O/E) ratio away from one indicates systematic overprediction and underprediction. We also generated flexible calibration plots [28]. The plot is a smooth curve (based on restricted cubic splines) of predicted risks from a secondary Cox model against the predicted risks from the model [16]. Finally, we performed a decision curve analysis to evaluate the clinical utility of the model in terms of net benefit [29]. Net benefit is a threshold-specific metric that is calculated as the proportion of true positives net of the weighted proportion of false positives, with the weights derived from the treatment threshold [29]. We compared the net benefit of the model to default strategies (i.e., treating all and treating none) at a clinically relevant range of treatment thresholds [30], specifically at 5%, 10% and 20%, given that a recent study has reported a threshold of 12% on 12-month AECOPD risk for low-dose azithromycin therapy [31]. Lower values were investigated to represent potential thresholds for inhaled medications (which have a better safety profile), while higher values represent thresholds for more expensive therapies such as biologics.

#### Sensitivity analysis

To reflect real-world community practice, NOVELTY included COPD patients as diagnosed by the physician. As a tool like ACCEPT is to be used in a similar setting, the primary analysis adhered to this definition. However, in a sensitivity analysis, we performed the same validation analysis (model recalibration and validation) by restricting the sample to COPD patients with spirometry-confirmed airflow obstruction (FEV1/FVC < 0.7) [2].

## Results

Table 1 summarises the baseline characteristics of the final sample, which consisted of 5,181 physician-diagnosed COPD patients from 18 countries: Argentina, Australia, Brazil, Canada, Colombia, Denmark, France, Germany, Italy, Japan, Mexico, the Netherlands, Norway, South Korea, Spain, Sweden, the UK and the USA. Country-specific sample sizes ranges from 39 in Norway to 976 in the USA. Overall, the average annual rates of moderate-to-severe and severe AECOPDs were, respectively, 0.58 (SD = 1.11) and 0.18 (SD = 0.51) episodes per person-year in the 12-month period prior to the index date. The average age was 66.5 (SD = 9.3) years, and 59.4% of the sample were male. Average BMI was 28.0 kg/m^2^ (SD = 6.6), 27.7% were current smokers (64.2% former smokers), and 14.9% had CVD. Most patients (62.8%) had moderate-to-severe COPD, with 39.5% classified as GOLD 2 and 23.3% as GOLD 3 severity grades. 59.8% had an mMRC dyspnoea score of 1–2. The proportion of missing values in these baseline characteristics was low in general, i.e., < 11% except for use of domiciliary oxygen therapy, with over 60% missingness (Additional file 1 Appendix Table A1). The comparison in baseline characteristics between the NOVELTY cohort and the recalibration cohort of ACCEPT 2.0, and the baseline characteristics of included patients from each participating country in NOVELTY, are, respectively, summarised in Additional file 1 Appendix Tables A2 and A3.

**Table 1:** Baseline characteristics of NOVELTY patient sample

Parameter	All (*N* = 5,181)	FEV_1_/FVC < 0.7 (*N* = 3,705)
Age measured in years, mean (SD)	66.5 (9.3)	67.2 (8.8)
Males, n(%)	3075 (59.4)	2316 (62.5)
Race group, n(%)		
Caucasian African American North East Asian South East Asian Other race group	4109 (79.3) 311 (6) 297 (5.7) 229 (4.4) 235 (4.5)	3031 (81.8) 137 (3.7) 205 (5.5) 176 (4.8) 156 (4.2)
BMI measured in kg/m^2^, mean (SD) Missing, n(%)	28.0 (6.6) 304 (5.9)	27.3 (6.2) 160 (4.3)
Smoking status, n(%)		
Current smoker Former smoker Never smoked Missing	1433 (27.7) 3324 (64.2) 408 (7.9) 16 (0.3)	967 (26.1) 2484 (67.0) 248 (6.7) 6 (0.2)
Smoking pack years, mean (SD)	39.9 (37)	42.4 (38.4)
FEV_1_ measured in litres, mean (SD) Missing, n(%)	1.6 (0.7) 348 (6.7)	1.5 (0.7) 0 (0)
FEV_1_ percent predicted, mean (SD) Missing, n(%)	60.6 (22.8) 544 (10.5)	55.0 (20.7) 148 (4.0)
FVC measured in litres, mean (SD) Missing, n(%)	2.9 (1) 361 (7)	2.9 (1.0) 2 (0.1)
FVC percent predicted, mean (SD) Missing, n(%)	82.1 (20.8) 557 (10.8)	82.5 (21.2) 150 (4.0)
Blood eosinophil count measured in 10^9^/litre, geometric mean (SD)	0.16 (1.93)	0.16 (1.90)
Missing, n(%)	2541 (49.04)	1730 (46.7)
GOLD severity^*^, n(%)		
1 2 3 4 Missing	949 (18.3) 2048 (39.5) 1206 (23.3) 434 (8.4) 544 (10.5)	436 (11.8) 1559 (42.1) 1139 (30.7) 423 (11.4) 148 (4.0)
Taking ICS, n(%)	2926 (56.5)	2186 (59.0)
Taking LABA, n(%)	3436 (66.3)	2625 (70.9)
Taking LAMA, n(%)	3109 (60)	2421 (65.3)
Cardiovascular comorbidity, n(%)	771 (14.9)	565 (15.2)
Domiciliary oxygen therapy, n(%) Missing	151 (2.9) 3352 (64.7)	131 (3.5) 3371 (61.3)
mMRC dyspnoea score, n(%)		
0 1 2 3 4 Missing	710 (13.7) 1754 (33.9) 1344 (25.9) 939 (18.1) 280 (5.4) 154 (3)	443 (12.0) 1248 (33.7) 1006 (27.2) 712 (19.2) 218 (5.9) 78 (2.1)
Rate of moderate-to-severe exacerbations at baseline, mean (SD)	0.58 (1.11)	0.60 (1.04)
Rate of severe exacerbations at baseline, mean (SD)	0.18 (0.51)	0.21 (0.54)
Rate of moderate-to-severe exacerbations during Year 1, mean (SD)	0.44 (1.00)	0.49 (1.03)
Rate of severe exacerbations during Year 1, mean (SD)	0.13 (0.49)	0.16 (0.52)
Average follow-up time in days, mean (SD)	356.8 (41.1)	357 (39.2)

*GOLD severity classification based on percent predicted forced expiratory volume in 1 s (FEV1P). GOLD 1: Mild (≥ 80%); GOLD 2: Moderate (50–79%); GOLD 3: Severe (30–49%); GOLD 4: Very Severe (< 30%)

Abbreviations: BEC: Blood Eosinophil Count; BMI: Body Mass Index; FEV_1_: Forced Expiratory Volume in 1 s; FEV_1_P: Forced Expiratory Volume in 1 s expressed in Percent Predicted; FVC: Forced Vital Capacity; FVCP: Forced Vital Capacity expressed in Percent Predicted; GOLD: Global Initiative for Chronic Obstructive Lung Disease; ICS: Inhaled Corticosteroid; LABA: long-acting beta agonist; LAMA: long-acting muscarinic receptor antagonist; mMRC: modified Medical Research Council; SD: Standard Deviation

**Fig. 2:**
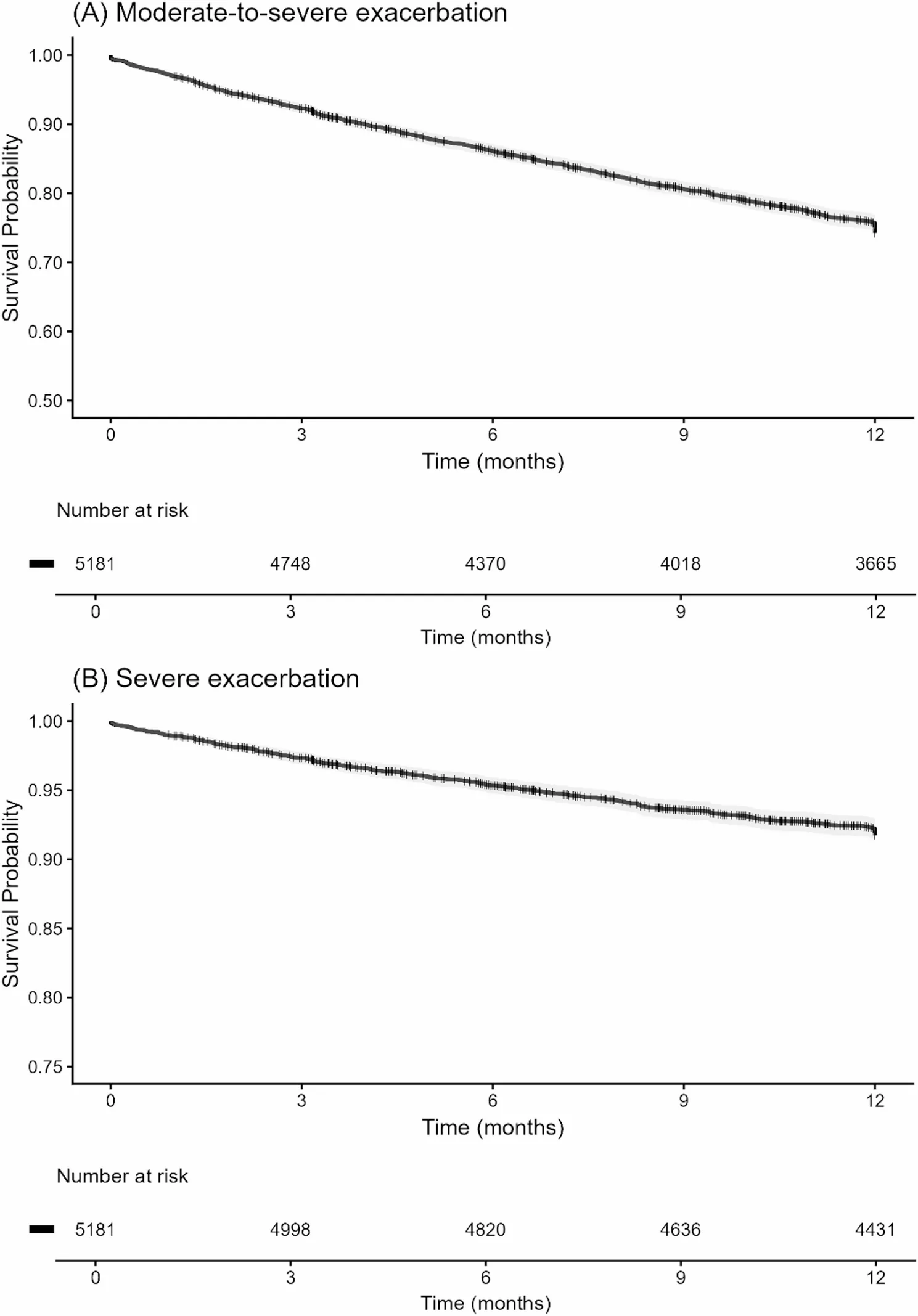
Displays the Kaplan-Meier survival curve of 12-month moderate-to-severe AECOPD (panel **A**) and severe AECOPD (panel **B**) in the NOVELTY sample. At 12 months, the Kaplan-Meier estimate of moderate-to-severe and severe AECOPD risks were 0.26 and 0.08, respectively

The mean follow-up time was 357 days (SD = 41). The 12-month observed risk of moderate-to-severe AECOPD varied widely, between 0.11 in South Korea and 0.46 in the UK and the Netherlands (overall mean = 0.26), whereas the observed severe AECOPD risk ranged from 0.034 in South Korea to 0.21 in Colombia (overall mean = 0.08). The Kaplan-Meier survival curves are presented in Fig. 2. Country-specific Kaplan-Meier survival curves and exacerbation rates are presented in Additional file 1 Appendix Figures A2 and A3.

### External validation of ACCEPT 2.0

**Fig. 3:**
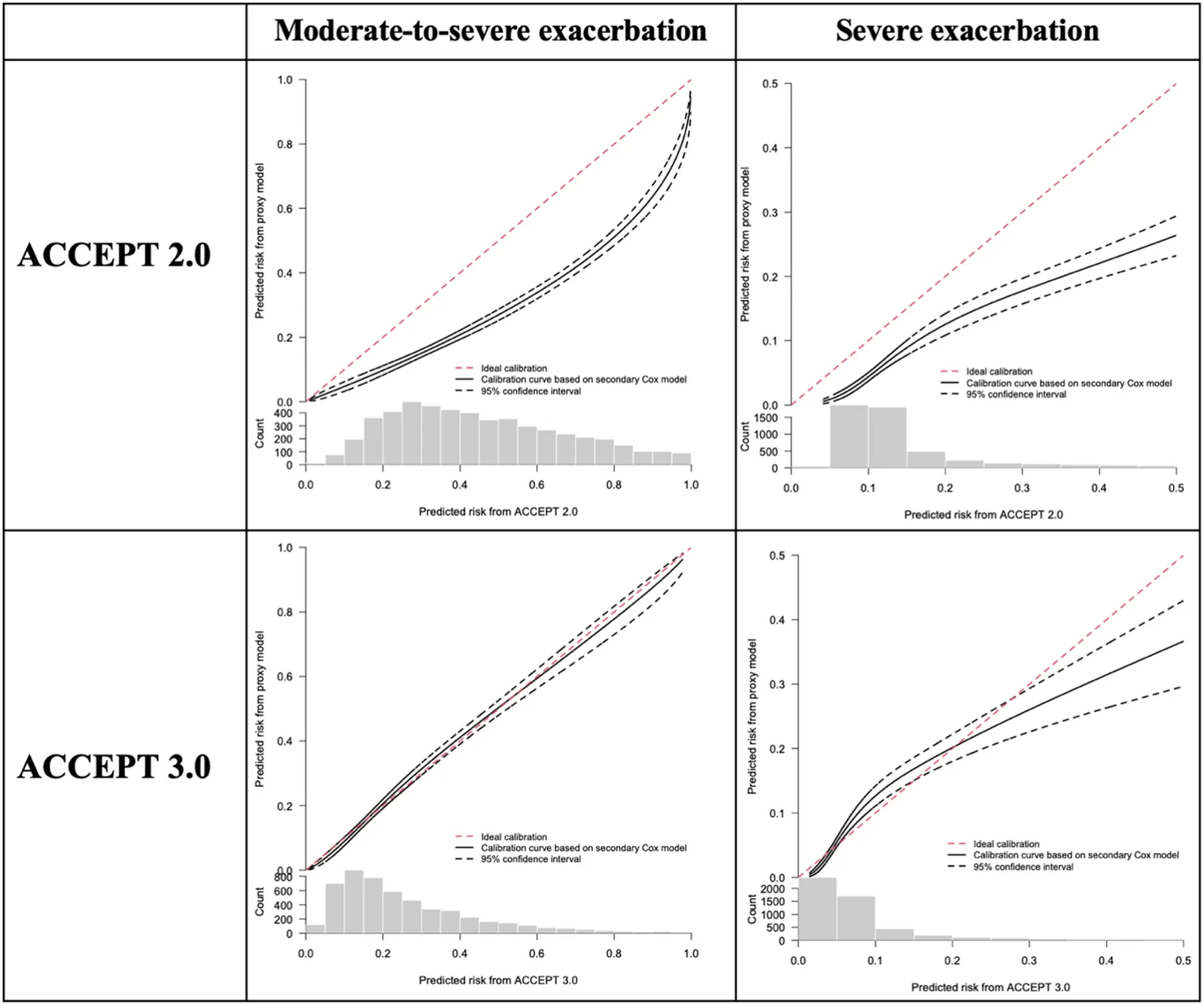
Displays the calibration plots of ACCEPT 2.0 and ACCEPT 3.0 in NOVELTY. The top panel shows the calibration of 12-month predicted risk of moderate-to-severe and severe COPD exacerbations in ACCEPT 2.0. The bottom panel shows the calibration of 12-month predicted risk of moderate-to-severe and severe COPD exacerbations in ACCEPT 3.0. The y-axes represent the observed risks estimated from a secondary Cox model. The x-axes represent the predicted risks from the model under inspection

The AUROC at 12 months was 0.73 (95% CI: 0.72–0.75) for moderate-to-severe AECOPD and 0.76 (95% CI: 0.73–0.78) for severe AECOPD. The AUROC curves are presented in Appendix Figure A4 in Additional file 1. The mean predicted risks (moderate-to-severe AECOPD: 0.45; severe AECOPD: 0.16) were substantially higher than the corresponding observed risks, with O/E ratios of 0.57 (95% CI: 0.54–0.60) and 0.51 (95% CI: 0.46–0.56), respectively. The calibration plots showed overprediction for both outcomes at the entire range of predicted risks (Fig. 3).

**Fig. 4:**
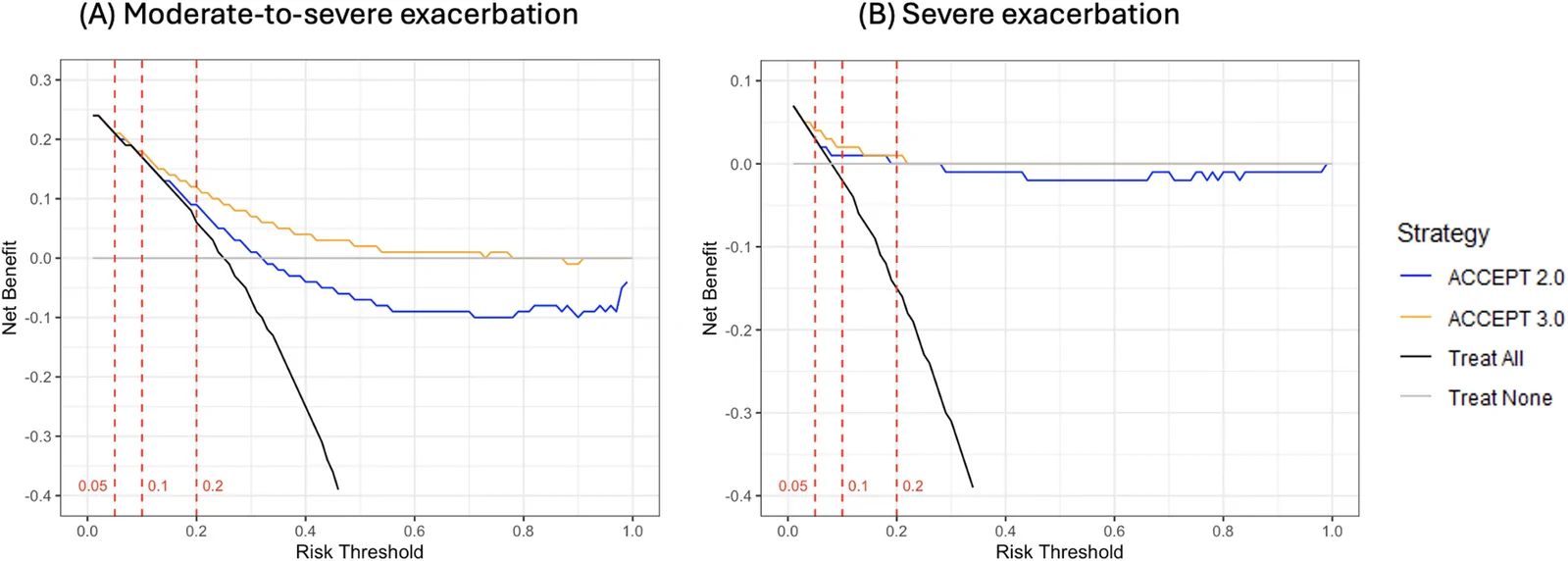
Displays the decision curves of ACCEPT 2.0 and ACCEPT 3.0 for moderate-to-severe AECOPD (panel A) and severe AECOPD (panel B). At the thresholds of 5%, 10% and 20%, ACCEPT 3.0 demonstrated a net benefit of 0.21, 0.18 and 0.12 for moderate-to-severe AECOPD, and 0.044, 0.020, and 0.006 for severe AECOPD, which were consistently higher than ACCEPT 2.0 (0.21, 0.17 and 0.085 for moderate-to-severe AECOPD, and 0.033, 0.008 and 0.003 for severe AECOPD), treating all (0.21, 0.17 and 0.064 for moderate-to-severe AECOPD; 0.003, -0.022 and − 0.15 for severe AECOPD) and treating none (0)

Despite such miscalibration, ACCEPT 2.0 still provided positive net benefit across a wide range of treatment thresholds (Fig. 4). At the thresholds of 5%, 10% and 20%, the net benefit was higher than treating all or treating none. However, ACCEPT 2.0 resulted in negative net benefit at thresholds > 0.33 for moderate-to-severe AECOPD and > 0.24 for severe AECOPD.

### ACCEPT 3.0: Recalibration of ACCEPT 2.0 with country-level adjustment

We recalibrated ACCEPT 2.0 with country-level random effects and summarised the model coefficients in Table 2. Compared to ACCEPT 2.0, ACCEPT 3.0 showed slightly improved discrimination with an AUROC of 0.75 (95% CI: 0.74–0.77) for moderate-to-severe AECOPD and 0.76 (95% CI: 0.73–0.78) for severe AECOPD (ROC curves shown in Additional file 1 Appendix Figure A4). The calibration, on the other hand, was substantially improved. The average predicted risks (0.26 and 0.08, respectively) were close to the observed risks, resulting in O/E ratios of 0.99 (95% CI: 0.94–1.05) and 0.99 (95% CI: 0.90–1.09) for the primary and secondary outcomes, respectively. Similarly, the calibration plots showed substantially improved calibration for both outcomes (Fig. 3; country-specific calibration plots are provided in Additional file 1 Appendix Figure A5).

**Table 2:** Model coefficients of ACCEPT 3.0

Parameter	Estimate, ln(HR)	*p*-value
**Predictor**		
Transformed predicted risk^*^		
Moderate-to-severe AECOPD Severe AECOPD	0.9120 0.9631	< 0.0001 < 0.0001
**Country-specific Intercept**		
Argentina	-0.1184	-
Australia	-0.1200	-
Brazil	-0.3274	-
Canada	-0.0337	-
Colombia	-0.1165	-
Germany	-0.4735	-
Denmark	0.0409	-
Spain	0.3034	-
France	-0.1859	-
United Kingdom	0.5697	-
Italy	0.3114	-
Japan	-0.4069	-
South Korea	-0.5942	-
Mexico	-0.2885	-
The Netherlands	0.4651	-
Norway	0.3277	-
Sweden	-0.0326	-
United States	-0.2881	-

^*^ Logarithm of the predicted rate (which is equivalent to the complementary log-log transformed predicted risk) from ACCEPT 2.0

Abbreviations: AECOPD: Acute Exacerbation of Chronic Obstructive Pulmonary Disease

In the decision curve analysis (Fig. 4), ACCEPT 3.0 showed consistently higher net benefit relative to ACCEPT 2.0 across the entire range of treatment thresholds, particularly for severe AECOPD. Table 3 presents a comparison of model performance between ACCEPT 2.0 and ACCEPT 3.0.

**Table 3:** Performance of ACCEPT 2.0 and ACCEPT 3.0 in NOVELTY

Evaluation metric	Moderate-to-severe AECOPD		Severe AECOPD	
	ACCEPT 2.0	ACCEPT 3.0	ACCEPT 2.0	ACCEPT 3.0
**Model Discrimination**				
Time-dependent AUROC at 12 months	0.73 (0.72–0.75)	0.75 (0.74–0.77)	0.76 (0.73–0.78)	0.76 (0.73–0.78)
**Model Calibration**				
O/E ratio Slope	0.57 (0.54–0.60) 0.98 (0.91–1.06)	0.99 (0.94–1.05) 1.01 (0.94–1.07)	0.51 (0.46–0.56) 0.96 (0.87–1.06)	0.99 (0.90–1.09) 1.02 (0.92–1.12)
**Model Net Benefit – ‘Treat All’ Net Benefit**				
5% threshold 10% threshold 20% threshold	0.21–0.21 = 0 0.17–0.17 = 0 0.085 − 0.064 = 0.021	0.21–0.21 = 0 0.18 − 0.17 = 0.01 0.12 − 0.064 = 0.056	0.033 − 0.003 = 0.03 0.008-(-0.022) = 0.03 0.003-(-0.15) = 0.153	0.044 − 0.003 = 0.041 0.020-(-0.022) = 0.042 0.006-(-0.15) = 0.156

Note: Time-dependent AUROC at 12 months and O/E ratio are presented as mean (95% confidence interval)

Abbreviations: AECOPD: Acute Exacerbation of Chronic Obstructive Pulmonary Disease; AUROC: Area Under the Receiver Operating Characteristic Curve; CI: Confidence Interval; O/E ratio: Observed-to-expected ratio

Title of data: Information on ACCEPT 3.0 and supplemental results

Description of data: This file presents an overview of the framework and the underlying data sources of ACCEPT, the algorithms for calculating ACCEPT 3.0 predictions from ACCEPT 2.0 and a graphical illustration of the approach to model recalibration that we adopted. The following supplemental results are included: country-specific Kaplan-Meier survival curve and rates of moderate-to-severe and severe AECOPD; receiver operating characteristic (ROC) curves of ACCEPT 2.0 and ACCEPT 3.0; country-specific calibration plots of ACCEPT 2.0 and ACCEPT 3.0; the relationship between country-specific observed risks and intercepts in ACCEPT 3.0; Kaplan-Meier survival curve of 12-month moderate-to-severe and severe AECOPD in COPD patients with FEV1/FVC < 0.7; AUROC curves, calibration plots and decision curves of ACCEPT 2.0 and ACCEPT 3.0 in sensitivity analysis

The full algorithm describing how predictions from ACCEPT 2.0 are modified for a country or setting with known background exacerbation risk is provided in Additional file 1 Appendix Box A1 and Box A2. Appendix Figure A6 in Additional file 1 further shows the linear relationship between country-specific observed risks and random effects in ACCEPT 3.0 (p-value of slope < 0.0001, adjusted R^2^ = 0.877) which provides an approximate method for estimating the country-level effect for any new country based on average 12-month AECOPD risk.

In a sensitivity analysis, we analysed patients with COPD diagnosed as FEV_1_/FVC < 0.7 (*n* = 3,705). The average annual rates of moderate-to-severe and severe AECOPD were, respectively, 0.60 (SD = 1.04) and 0.21 (SD = 0.54) episodes per year. The mean length of follow-up was 357 days (SD = 39), and the mean observed risks of moderate-to-severe and severe AECOPD were 0.29 and 0.096 respectively (Kaplan-Meier survival curves are presented in Additional file 1 Appendix Figure A7). Comparing the models before and after recalibration, the AUROCs at 12 months were, respectively, 0.73 (95% CI: 0.71–0.75) and 0.75 (95% CI: 0.73–0.77) for moderate-to-severe AECOPD, and 0.75 (95% CI: 0.72–0.78) and 0.75 (95% CI: 0.73–0.78) for severe AECOPD (AUROC curves are presented in Additional file 1 Appendix Figure A8). Similar to the results of the main analysis, calibration and net benefit were both enhanced from model recalibration (Additional file 1 Appendix Figures A9 and A10).

## Discussion

In this study, we recalibrated ACCEPT 2.0 to account for country-specific background exacerbation risk using multi-country observational data of NOVELTY. The original ACCEPT 2.0 demonstrated good discrimination in NOVELTY but systematically overpredicted risks at all levels of predicted risks. This likely reflected the fact that ACCEPT was developed in a highly enriched samples for AECOPD, thereby overestimating the risk in a real-world sample such as NOVELTY. Recalibrating ACCEPT 2.0 with an overall slope-correction factor and country-specific random effects resulted in significant improvement in model calibration. Importantly, ACCEPT 3.0 demonstrated consistently higher net benefit for both moderate-to-severe and severe AECOPD, especially for the latter, at the 5%-20% risk thresholds which seem to be the plausible range of treatment thresholds for clinical decision-making. ACCEPT 3.0 tended to overestimate the risk at higher predicted risks for severe events, but we note that there were very few observations in this range. To extend the applicability of ACCEPT 3.0 to non-NOVELTY settings, we showed that country-specific random effects can be approximately linearly modelled as a function of the average observed risk within each setting. For example, a hospital system can estimate the AECOPD risk from their previous-year records and use the algorithm detailed in this paper to estimate their local intercept term for ACCEPT 3.0. In addition to providing in this paper a step-by-step guide on updating risk scores obtained from ACCEPT 2.0, we also made ACCEPT 3.0 available on a publicly available webapp (https://resp.core.ubc.ca/ipress/accept) and a web-based Application Programming Interface [32].

We documented significant heterogeneity in AECOPD frequency among participating countries in the NOVELTY study, with a five-fold difference in annualised rates over follow-up. This could reflect variations in patient-level exacerbation risk (e.g., due to demographic characteristics, treatment protocols, environmental exposure), data collection procedures (e.g., completeness of exacerbation date information, predictor assessment, documentation of exacerbation history), and outcome assessment across countries, though these effects could not be disentangled with the available variables in the NOVELTY study. Such wide variation in risks warranted localised recalibration of the model. Notably, previous COPD trials and epidemiological studies [14, 15, 33] have shown that between-setting heterogeneity in COPD risks cannot be fully explained by salient predictors. As such, while relative predictor effects might remain stable across populations, the absolute risks can substantially vary. Variability in outcome risks is also observed for ‘harder’ clinical outcomes such as major adverse CVD events, resulting in popular risk scoring tools such as Framingham risk score having substantial variations across settings and populations [34]. For a subjective outcome such as AECOPD that is more dependent on patients’ experience of symptoms, such variations might be even greater. Overall, these results indicate that updating AECOPD prediction algorithms based on local estimates of outcome risk when transporting them from one setting to another might be necessary to retain calibration and clinical utility.

The inclusion of system-level factors in the next generation of risk scoring algorithms can potentially explain some of the observed heterogeneity. The seasonality of AECOPD, with evidence showing a marked increase in incidence during winter months [35], indicates that climate-related factors are worth considering. The actual causes of the seasonality have not been identified, but are possibly linked to more prevalent triggers, especially respiratory viral infections in cold and damp environments [35]. Most respiratory viral infections showed seasonal patterns, with high incidence during winter in temperate regions and during the rainy season in tropical regions [36, 37]. Such information can plausibly be incorporated into risk scoring tools. Considering the interacting effects of seasonality, latitude and CVD comorbidity risks, the combined effects of geographical region and temporal seasonality on AECOPD risk should be further accounted for in clinical risk prediction. System-level factors such as access to treatments and local health system capacity can also be considered, albeit their variability across settings within the same region might indicate that reliable information on these factors is less available compared with metrics related to the climate and environment.

Multivariable risk prediction models such as ACCEPT, the Bertens model, and the Birmingham Lung Improvement Studies score have demonstrated superior predictive performance as compared to exacerbation history as a marker for future exacerbations, but setting-specific recalibration is required for enhanced clinical utility [38, 39]. Our parsimonious updating strategy (one set of country-specific intercepts and a fixed correction slope) retains much of the learning from the previous development samples while adjusting the model for new settings [10]. The inclusion of a random intercept extends the generalisability of ACCEPT 3.0 to settings within and beyond the validation sample, as local AECOPD prevalence can be mapped to a new estimate of random intercept. Such updating improved model calibration, while discrimination remains largely unchanged as these methods do not affect the relative ranking of the predicted risks [10]. To achieve better discriminatory power, more complex techniques are required, and this could range from adjustment of predictor weights to adding a new predictor to the existing model [10]. However, ACCEPT 2.0 already showed good discrimination, and such updates should also consider the usability of a model with many predictors.

Current paradigms for AECOPD risk stratification are mostly based on exacerbation history, with the most common classifications considering the occurrence of ≥ 1 event in the previous 12 months as an indicator of being ‘high risk’ for future AECOPDs [2]. Such qualitative classifications that do not attach a numerical estimate of risk to low- and high-risk categories do not appropriately take into account the heterogeneity in background risk across settings, resulting in potential misclassification of risk [40]. On the other hand, a quantitative risk-based approach can provide mechanisms for correcting for such differences in risk. As such, being able to include a local estimate of AECOPD risk to make valid predictions should be taken as an advantage of using risk scoring algorithms compared with qualitative stratification based on AECOPD history. With demonstrated superior net benefit relative to ACCEPT 2.0, ACCEPT 3.0 could be used to complement guideline-based risk stratification with a quantitative estimate of risk that is likely to provide higher clinical utility. Of note, treatment effect estimates have also been integrated into the ACCEPT webapp to guide personalised preventive treatment (e.g., for roflumilast and azithromycin) [5].

Limitations of this study should be acknowledged. Exacerbation history and COPD treatment during the pre-index 12-month period were assessed by patient recall. However, many clinical practices now use electronic medical records which can more objectively verify such information. Second, the low sample size in some participating countries (Denmark = 51, Mexico = 58, Norway = 39) may result in high levels of uncertainty in country-specific corrections for those settings. This could be the driving factor of the poor setting-specific calibration observed for small-sample or low-count countries in our cohort. To address this, we focused on overall model performance across all countries. However, recalibrated scores may be expected to perform well in the data used for recalibration. In addition, while a score algorithm for settings not covered by the NOVELTY study is provided, the modified regression coefficients are based only on the countries in this study. As such, we recommend dedicated, country-specific validation studies as the next step. Third, the possibility of missing-not-at-random among predictors cannot be fully excluded, and the imputation of incomplete information in exacerbation dates using the latest possible date could result in misclassification of outcome. However, in this study, earliest-date imputation yielded near-identical estimates. In addition, the recruitment of patients who were more likely to complete a three-year follow-up into the NOVELTY study could also have introduced a selection bias that resulted in a healthier patient cohort. In general, in advanced COPD, exacerbation risk might have a different relationship with patient characteristics, demanding separate risk assessment and model development for such patients. Fourth, our analysis did not account for competing risk of death (albeit it was low, at 1.8% of the analysis sample – thus unlikely to have a measurable effect on model performance) nor time-varying effects (e.g., changing treatments over time). Future work should aim to incorporate these aspects. Of note, as we made predictions for 12 months, the effect of seasonality likely cancels out in our predictions. However, future models that aim at predicting shorter-term risks need to consider seasonality. Future updating of ACCEPT could also consider the inclusion of other candidate predictors such as blood eosinophil count [41], which was excluded during the development of ACCEPT due to the general unavailability of such blood biomarkers in clinical settings.

In conclusion, the newly derived ACCEPT 3.0, which is based on recalibration of ACCEPT 2.0 with country-specific intercepts and a fixed slope, yielded substantially improved calibration and consistently higher net benefit. Background exacerbation risks should be accounted for when adjusting predicted exacerbation risks in a new setting.

## Supplementary Information

Below is the link to the electronic supplementary material.

Supplementary Material 1

## Data Availability

The data that support the findings of this study are available from NOVELTY but restrictions apply to the availability of these data, which were used under license for the current study, and so are not publicly available. A study protocol was developed as a student project to obtain access to the data from the provider. It was not meant for publication but is available upon request. The code used for data analyses is available from https://github.com/resplab/papercode/tree/main/ACCEPT3.0.
