# Supplementary material for: ACCEPT 3.0: Recalibrating ACute COPD Exacerbation Prediction Tool (ACCEPT) for global use
Source: Diagn Progn Res. 2026 Oct 8;10:27. doi: 10.1186/s41512-026-00242-x (PMC13651374; doi:10.1186/s41512-026-00242-x)
Supplement: Supplementary file 1 [file 41512_2026_242_MOESM1_ESM.pdf]

ACCEPT 3.0: Recalibrating ACute COPD Exacerbation Prediction Tool (ACCEPT) for Global Use – Supplementary Material

Table of Contents

Appendix 1: ACCEPT framework and underlying data sources 2

Appendix Box 1: Algorithms for calculating ACCEPT 3.0 predictions from ACCEPT 2.0 3

Appendix Box 2: Examples of calculating ACCEPT 3.0 predictions from ACCEPT 2.0 4

Appendix Figure A1: Graphical illustration of model recalibration 6

Appendix Table A1:Original and imputed baseline characteristics of NOVELTY cohort 7

Appendix Table A2: Baseline characteristics of ACCEPT 2.0 recalibration cohort (ECLIPSE) vs ACCEPT 3.0 recalibration cohort (NOVELTY) 9

Appendix Table A3: Baseline characteristics of NOVELTY cohort by country....10

Appendix Figure A2: Country-specific Kaplan-Meier survival curve....12

Appendix Figure A3: Country-specific rates of moderate and severe AECOPD 13

Appendix Figure A4: ROC curves of ACCEPT 2.0 and ACCEPT 3.0....14

Appendix Figure A5: Country-specific calibration plots of ACCEPT 2.0 and ACCEPT 3.0....15

Appendix Figure A6: Relationship between country-specific observed risk and intercept in ACCEPT 3.0....19

Appendix Figure A7: Kaplan-Meier survival curve of 12-month moderate-to-severe and severe AECOPD in COPD patients with $FEV_1/FVC < 0.7$ 20

Appendix Figure A8: ROC curves of ACCEPT 2.0 and ACCEPT 3.0 in COPD patients with $FEV_1/FVC < 0.7$.21

Appendix Figure A9: Calibration plots of ACCEPT 2.0 and ACCEPT 3.0 in COPD patients with $FEV_1/FVC <$ 0.7....22

Appendix Figure A10: Decision curves of ACCEPT 2.0 and ACCEPT 3.0 in COPD patients with $FEV_1/FVC <$ 0.7....23

References....24

Appendix 1: ACCEPT framework and underlying data sources

In the development of ACCEPT, Adibi and colleagues characterised the rate and severity of exacerbations using a joint accelerated failure time and logistic model.[1] In brief, this approach assigns two random-effect terms to each individual, measuring their rate of exacerbation and the likelihood that once exacerbation occurs, it will be severe. This method enables the hazard (and severity) of all exacerbations at any follow-up timepoint to be specified for each patient, allowing different predictions such as the risk of experiencing a specific number of total and severe exacerbations over the next 12 months.

ACCEPT was developed using data from patients with COPD, with no prior or existing history of asthma, and who had at least one exacerbation over the past year. Data was pooled from all groups of three randomised controlled trials:

1. Macrolide Azithromycin to Prevent Rapid Worsening of Symptoms Associated With COPD (MACRO),[2] which assessed the effect of daily low-dose azithromycin therapy on exacerbation rates in patients with COPD. The data (study period: March 2006-June 2010, 1 year) covered 17 sites in the USA and included patients with the following criteria: aged >40 years, had a clinical diagnosis of COPD, had at least 10 pack-years of smoking, oxygen or systemic glucocorticoids therapy in the past year, emergency visit or admission to hospital. (n=1,107)

2. Simvastatin in the Prevention of COPD Exacerbations (STATCOPE),[3] which assessed effects of daily simvastatin therapy on exacerbation rates. The data (study period: March 2010-January 2014, about 2 years) covered 45 sites across North America and included patients with the following criteria: aged 40–80 years, had a clinical diagnosis of COPD, had at least 10 pack-years of smoking, receiving supplemental oxygen or treatment with glucocorticoids or antibiotics, or emergency visit or admission to hospital in the past year. (n=847)

3. Optimal Therapy of COPD to Prevent Exacerbations and Improve Quality of Life (OPTIMAL),[4] which assessed effects of tiotropium + fluticasone + salmeterol on exacerbation rates compared with tiotropium + fluticasone, and tiotropium alone. The data (study period: October 2003-January 2006, 1 year) covered 27 sites in Canada and included patients with the following criteria: >35 years, had a clinical diagnosis of COPD, had at least 10 pack-years of smoking, exacerbation requiring systemic glucocorticoids or antibiotics therapy in the past year. (n=426)

While the developmental data did not include patients with no exacerbation in the previous year, ACCEPT demonstrated in an external validation the ability to generate robust predictions for such patients. Of note, the development data also did not include patients with mild (GOLD 1) severity, patients with COPD with a history of asthma, lifetime non-smokers, patients <40 years or >80 years, and populations not within North America, Europe, or Oceania, and hence the generalisability of ACCEPT predictions for these subgroups has not been established yet.

ACCEPT was recalibrated to ACCEPT 2.0 using data from Evaluation of COPD Longitudinally to Identify Predictive Surrogate End-points (ECLIPSE)[5], an independent 3-year observational COPD cohort study from December 2005 to February 2010, primarily conducted to characterise COPD phenotypes and identify novel markers of disease progression. The data covered 46 sites across 12 countries and included patients regardless of exacerbation history who were aged 40–75 years, had a clinical diagnosis of COPD, had >10 pack-years of smoking with no less than 0.3 years of follow-up. (n=1,803)

Appendix Box 1: Algorithms for calculating ACCEPT 3.0 predictions from ACCEPT 2.0

Box 1: Step-by-step algorithm for estimating recalibrated 12-month risk of moderate-to-severe or severe AECOPD from ACCEPT 2.0

1. **Estimate the country-specific intercept term for your local setting.**
   - If your country participated in NOVELTY, or you consider your local setting similar to the settings participating in NOVELTY, then pick the intercept term from ***Table 2***
   - If your country is not listed in ***Table 2***, or you consider your setting not to be representative, you can estimate the intercept from average 12-month AECOPD risk from your setting via the following equation:

     Intercept = 3.102 x [average 12-month moderate-to-severe AECOPD risk] - 0.867

2. **Obtain predicted 12-month rate of AECOPD from ACCEPT 2.0.** The webapp of ACCEPT 2.0 (available at https://resp.core.ubc.ca/ipress/accept) allows users to enter a pre-specified patient's demographic and clinical characteristics and it returns a predicted rate estimate as output.

3. **Update predicted rates**

   Update rate = exp(-0.8107 + Intercept + 0.9120 * ln(Predicted Rate)) (for moderate-to-severe AECOPD risk)

   Update rate = exp(-0.8304 + Intercept + 0.9631*ln(Predicted Rate)) (for severe AECOPD risk)

4. **Convert the rate estimate to risk.** using the formula:

   Update risk = 1-exp(-Updated rate)

Note: The outcome rate is related to the outcome risk by the formula: rate = -log(1-risk).

Appendix Box 2: Examples of calculating ACCEPT 3.0 predictions from ACCEPT 2.0

<u>Hypothetical Patient Profile</u>

- Gender: Female
- Age (years): 45
- Current smoker: No
- Post-bronchodilation FEV1 (% predicted): 75
- Modified Medical Research Council (mMRC) score: 3
- Body mass index (BMI): 24
- Use of oxygen therapy within the last year: No
- Currently on statin: Yes
- Currently on LAMAs: No
- Currently on LABAs: No
- Currently on inhaled corticosteroids: Yes
- Number of all exacerbations within the last 12 months: 1
- Number of severe exacerbations within the last 12 months: 0

Box 2A: Estimating recalibrated 12-month risk of **severe** AECOPD from ACCEPT 2.0 assuming the hypothetical patient is from **Canada (a participating country in the NOVELTY study)**

1. **Estimate the country-specific intercept term.** Since Canada is a participating country in the NOVELTY study, pick the intercept term from ***Table* 2:** Intercept = -0.0337.

2. **Obtain predicted 12-month <u>rate</u> of severe AECOPD from ACCEPT 2.0.** Enter the above-described hypothetical patient's information into the webapp of ACCEPT 2.0 (available at https://resp.core.ubc.ca/ipress/accept). Predicted 12-month rate of severe AECOPD = 0.1428.

3. **Update predicted rates**

   Update rate = exp(-0.8304 + Intercept + 0.9631*ln(Predicted Rate)) = exp(-0.8304 - 0.0337 + 0.9631*ln(0.1428)) = 0.0647

4. **Convert the rate estimate to risk.** using the formula:

   Update risk = 1-exp(-Updated rate) = 1-exp(-0.0647) = **<u>0.0626</u>**.

Box 2B: Estimating recalibrated 12-month risk of **moderate-to-severe** AECOPD from ACCEPT 2.0 assuming the hypothetical patient is from **Singapore (not a participating country in the NOVELTY study)**

1. **Estimate the country-specific intercept term.** Since Singapore is not a participating country in the NOVELTY study, estimate Singapore's intercept from its average 12-month AECOPD risk. In

Singapore, the average 12-month rate of moderate-to-severe AECOPD is an estimated 0.99,[6] which is equivalent to a 12-month risk of 1-exp(-0.99) = 0.628

Intercept = 3.102 x [average 12-month moderate-to-severe AECOPD risk] - 0.867 = 3.102 x 0.628 - 0.867 = 1.081

2. **Obtain predicted 12-month rate of moderate-to-severe AECOPD from ACCEPT 2.0.** Enter the above-described hypothetical patient's information into the webapp of ACCEPT 2.0 (available at https://resp.core.ubc.ca/ipress/accept). Predicted rate of moderate-to-severe AECOPD = 1.2057.

3. **Update predicted rates**

   Update rate = exp(-0.8107 + Intercept + 0.9120*ln(Predicted Rate)) = exp(-0.8107 + 1.081 + 0.9120*ln(1.2057)) = 1.5541

4. **Convert the rate estimate to risk.** using the formula:

   Update risk = 1-exp(-Updated rate) = 1-exp(-1.5541) = **0.7886**

Appendix Figure A1: Graphical illustration of model recalibration

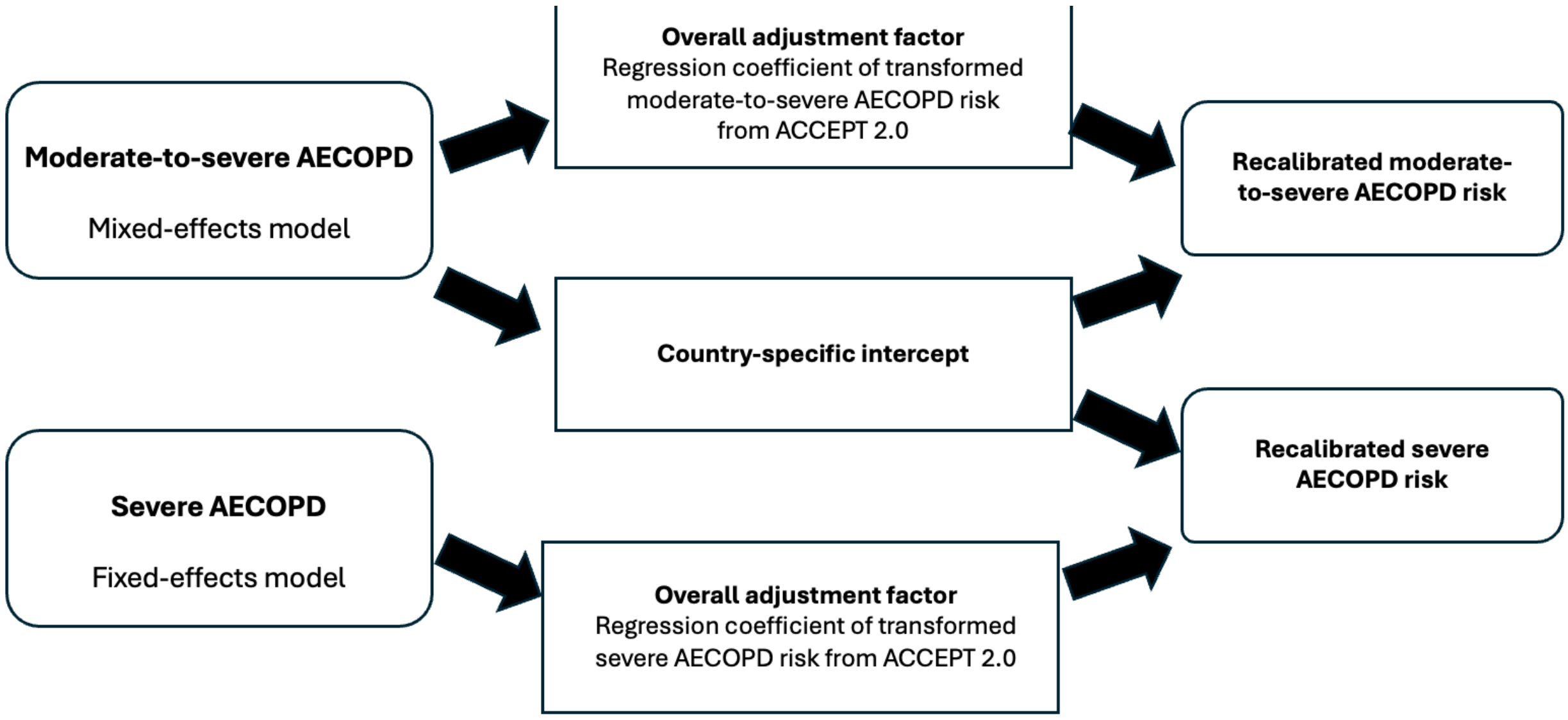

Appendix Table A1:Original and imputed baseline characteristics of NOVELTY cohort

Parameter	Main analysis (All, N=5,181)		Sensitivity analysis (FEV_1/FVC < 0.7, N = 3,705)	
	Original data	Imputed data	Original data	Imputed data
Age measured in years, mean (SD)	66.5 (9.3)	66.5 (9.3)	67.2 (8.8)	67.2 (8.8)
Males, n(%)	3075 (59.4)	3075 (59.4)	2316 (62.5)	2316 (62.5)
Race group, n(%)				
Caucasian	4109 (79.3)	4109 (79.3)	3031 (81.8)	3031 (81.8)
African American	311 (6)	311 (6)	137 (3.7)	137 (3.7)
North East Asian	297 (5.7)	297 (5.7)	205 (5.5)	205 (5.5)
South East Asian	229 (4.4)	229 (4.4)	176 (4.8)	176 (4.8)
Other race group	235 (4.5)	235 (4.5)	156 (4.2)	156 (4.2)
BMI measured in kg/m^2, mean (SD)	28 (6.6)	28 (6.5)	27.3 (6.2)	27.3 (6.2)
Missing/NAs, n(%)	304 (5.9)	0 (0)	160 (4.3)	0 (0)
Smoking status, n(%)				
Current smoker	1433 (27.7)	1438 (27.8)	967 (26.1)	968 (26.1)
Former smoker	3324 (64.2)	3325 (64.2)	2484 (67.0)	2485 (67.1)
Never smoked	408 (7.9)	418 (8.1)	248 (6.7)	252 (6.8)
Missing/NAs	16 (0.3)	0 (0)	6 (0.2)	0 (0)
Smoking pack years, mean (SD)	39.9 (37)	39.9 (37)	42.4 (38.4)	42.4 (38.4)
FEV_1 measured in litres, mean (SD)	1.6 (0.7)	1.6 (0.7)	1.5 (0.7)	1.5 (0.7)
Missing/NAs, n(%)	348 (6.7)	0 (0)	0 (0)	0 (0)
FEV_1 percent predicted, mean (SD)	60.6 (22.8)	60.8 (22.8)	55.0 (20.7)	54.9 (20.7)
Missing/NAs, n(%)	544 (10.5)	0 (0)	148 (4.0)	0 (0)
FVC measured in litres, mean (SD)	2.9 (1.0)	2.9 (1.0)	2.9 (1.0)	2.9 (1.0)
Missing/NAs, n(%)	361 (7)	0 (0)	2 (0.1)	0 (0)
FVC percent predicted, mean (SD)	82.1 (20.8)	82 (20.8)	82.5 (21.2)	82.3 (21.1)
Missing/NAs, n(%)	557 (10.8)	0 (0)	150 (4.0)	0 (0)
BEC measured in 10^9/litre, geometric mean (SD)	0.16 (1.93)	0.15 (0.91)	0.16 (1.9)	0.16 (1.9)
Missing/NAs, n(%)	2541 (49.04)	0 (0)	1730 (46.7)	0 (0)
GOLD, n(%)				
1	949 (18.3)	1093 (21.1)	436 (11.8)	452 (12.2)
2	2048 (39.5)	2269 (43.8)	1559 (42.1)	1617 (43.6)
3	1206 (23.3)	1343 (25.9)	1139 (30.7)	1188 (32.1)
4	434 (8.4)	476 (9.2)	423 (11.4)	448 (12.1)
Missing/NAs	544 (10.5)	0 (0)	148 (4.0)	0 (0)
Taking ICS, n(%)	2926 (56.5)	2926 (56.5)	2186 (59.0)	2186 (59.0)
Taking LABA, n(%)	3436 (66.3)	3436 (66.3)	2625 (70.9)	2625 (70.9)

Taking LAMA, n(%)	3109 (60)	3109 (60)	2421 (65.3)	2421 (65.3)
Cardiovascular comorbidity, n(%)	771 (14.9)	771 (14.9)	565 (15.2)	565 (15.2)
Domiciliary oxygen therapy, n(%)<br><br>Missing/NAs, n(%)	151 (2.9)<br><br>3352 (64.7)	406 (7.8)<br><br>0 (0)	131 (3.5)<br><br>3371 (61.3)	284 (7.7)<br><br>0 (0)
mMRC dyspnoea score, n(%)<br><br>0<br>1<br>2<br>3<br>4<br>Missing/NAs	 <br>710 (13.7)<br>1754 (33.9)<br>1344 (25.9)<br>939 (18.1)<br>280 (5.4)<br>154 (3)	 <br>719 (13.9)<br>1791 (34.6)<br>1407 (27.2)<br>980 (18.9)<br>284 (5.5)<br>0 (0)	 <br>443 (12.0)<br>1248 (33.7)<br>1006 (27.2)<br>712 (19.2)<br>218 (5.9)<br>78 (2.1)	 <br>446 (12.0)<br>1272 (34.3)<br>1036 (28.0)<br>732 (19.8)<br>219 (5.9)<br>0 (0)
Rate of moderate-to-severe exacerbations at baseline, mean (SD)	0.58 (1.11)	0.58 (1.11)	0.60 (1.04)	0.60 (1.04)
Rate of severe exacerbations at baseline, mean (SD)	0.18 (0.51)	0.18 (0.51)	0.21 (0.54)	0.21 (0.54)
Rate of moderate-to-severe exacerbations during Year 1, mean (SD)	0.44 (1.00)	0.44 (1.00)	0.49 (1.03)	0.49 (1.03)
Rate of severe exacerbations during Year 1, mean (SD)	0.13 (0.49)	0.13 (0.49)	0.16 (0.52)	0.16 (0.52)
Average follow-up time in days, mean(SD)	356.8 (41.1)	356.8 (41.1)	357 (39.2)	357 (39)

*GOLD severity classification based on percent predicted forced expiratory volume in 1 second (FEV1P). GOLD 1: Mild (≥80%); GOLD 2: Moderate (50–79%); GOLD 3: Severe (30–49%); GOLD 4: Very Severe (<30%).

Abbreviations: BEC: Blood Eosinophil Count; BMI: Body Mass Index; FEV_1: Forced Expiratory Volume in 1 Second; FEV_1P: Forced Expiratory Volume in 1 Second expressed in Percent Predicted; FVC: Forced Vital Capacity; FVCP: Forced Vital Capacity expressed in Percent Predicted; GOLD: Global Initiative for Chronic Obstructive Lung Disease; ICS: Inhaled Corticosteroid; LABA: long-acting beta agonist; LAMA: long-acting muscarinic receptor antagonist; mMRC: modified Medical Research Council; SD: Standard Deviation.

Appendix Table A2: Baseline characteristics of ACCEPT 2.0 recalibration cohort (ECLIPSE) vs ACCEPT 3.0 recalibration cohort (NOVELTY)

Parameter	ECLIPSE (N=1,803)	NOVELTY (N=5,181)
Age measured in years, mean (SD)	63.3 (7)	66.5 (9.3)
Males, n(%)	1175 (65.2)	3075 (59.4)
BMI measured in kg/m^2, mean (SD)	26.5 (5.8)	28.0 (6.6)
Current smoker, n(%)	494 (27.4)	1433 (27.7)
FEV_1 percent predicted, mean (SD)	48.5 (16.4)	60.6 (22.8)
Taking ICS, n(%)	1290 (71.5)	2926 (56.5)
Taking LABA, n(%)	1227 (68.1)	3436 (66.3)
Taking LAMA, n(%)	1279 (70.9)	3109 (60)
Cardiovascular comorbidity, n(%)	426 (23.6)	771 (14.9)
Domiciliary oxygen therapy, n(%)	124 (6.9)	151 (2.9)
Follow-up time in years, mean (SD)	0.98 (0.1)	0.99 (0.1)
Annual rate of moderate-to-severe exacerbations, mean (SD)	1.20	0.58 (1.11)
Annual rate of severe exacerbations, mean (SD)	0.27	0.18 (0.51)

Appendix Table A3: Baseline characteristics of NOVELTY cohort by country

Parameter	All	ARG	AUS	BRA	CAN	COL	DEU	DNK	ESP	FRA	GBR	ITA	JPN	KOR	MEX	NLD	NOR	SWE	USA
N	5181	232	378	106	501	186	345	51	449	244	499	245	297	210	58	184	39	181	976
Age measured in years, mean (SD)	66.5 (9.3)	64.8 (8.4)	67.9 (9.1)	68.7 (8.5)	68.7 (9.0)	70.9 (8.9)	65.1 (8.6)	64.4 (8.2)	65.2 (8.7)	65.1 (10.3)	66.3 (9.3)	67.1 (9.4)	70.9 (7.7)	65.5 (9.1)	68.5 (9.3)	66.0 (8.1)	66.8 (8.1)	70.4 (6.7)	63.6 (9.7)
Males, n(%)	3075 (59.4)	138 (59.5)	180 (47.6)	67 (63.2)	269 (53.7)	107 (57.5)	178 (51.6)	20 (39.2)	334 (74.4)	155 (63.5)	263 (52.7)	166 (67.8)	265 (89.2)	194 (92.4)	32 (55.2)	102 (55.4)	21 (53.8)	80 (44.2)	504 (51.6)
Race group - Caucasian, n(%)	4109 (79.3)	230 (99.1)	365 (96.6)	87 (82.1)	465 (92.8)	104 (55.9)	344 (99.7)	51 (100.0)	447 (99.6)	219 (89.8)	493 (98.8)	245 (100.0)	0 (0.0)	0 (0.0)	29 (50.0)	176 (95.7)	39 (100.0)	180 (99.4)	635 (65.1)
Race group - African American, n(%)	311 (6.0)	0 (0.0)	0 (0.0)	16 (15.1)	3 (0.6)	1 (0.5)	0 (0.0)	0 (0.0)	0 (0.0)	2 (0.8)	1 (0.2)	0 (0.0)	0 (0.0)	0 (0.0)	0 (0.0)	1 (0.5)	0 (0.0)	0 (0.0)	287 (29.4)
Race group - North East Asian, n(%)	297 (5.7)	0 (0.0)	0 (0.0)	0 (0.0)	0 (0.0)	0 (0.0)	0 (0.0)	0 (0.0)	0 (0.0)	0 (0.0)	0 (0.0)	0 (0.0)	297 (100.0)	0 (0.0)	0 (0.0)	0 (0.0)	0 (0.0)	0 (0.0)	0 (0.0)
Race group - South East Asian, n(%)	229 (4.4)	0 (0.0)	0 (0.0)	0 (0.0)	18 (3.6)	0 (0.0)	0 (0.0)	0 (0.0)	0 (0.0)	0 (0.0)	3 (0.6)	0 (0.0)	0 (0.0)	204 (97.1)	0 (0.0)	1 (0.5)	0 (0.0)	0 (0.0)	3 (0.3)
Race - Other race group, n(%)	235 (4.5)	2 (0.9)	13 (3.4)	3 (2.8)	15 (3.0)	81 (43.5)	1 (0.3)	0 (0.0)	2 (0.4)	23 (9.4)	2 (0.4)	0 (0.0)	0 (0.0)	6 (2.9)	29 (50.0)	6 (3.3)	0 (0.0)	1 (0.6)	51 (5.2)
BMI measured in kg/m^2, mean (SD)	28.0 (6.6)	28.7 (7.0)	29.2 (6.7)	26.6 (6.7)	29.4 (7.2)	27.2 (5.7)	26.9 (5.6)	26.1 (6.0)	28.4 (5.1)	27.1 (6.3)	28.3 (6.6)	27.8 (5.4)	23.2 (3.6)	23.6 (3.1)	28.7 (6.1)	27.5 (5.1)	25.9 (4.9)	26.8 (5.1)	30.1 (7.8)
BMI - Missing/NAs, n(%)	304 (5.9)	2 (0.9)	11 (2.9)	1 (0.9)	125 (25.0)	2 (1.1)	5 (1.4)	0 (0.0)	10 (2.2)	6 (2.5)	2 (0.4)	4 (1.6)	60 (20.2)	7 (3.3)	0 (0.0)	36 (19.6)	0 (0.0)	2 (1.1)	31 (3.2)
Smoking status - Current smoker, n(%)	1433 (27.7)	55 (23.7)	69 (18.3)	17 (16.0)	162 (32.3)	5 (2.7)	101 (29.3)	17 (33.3)	132 (29.4)	66 (27.0)	156 (31.3)	55 (22.4)	47 (15.8)	48 (22.9)	3 (5.2)	36 (19.6)	21 (53.8)	34 (18.8)	409 (41.9)
Smoking status - Former smoker, n(%)	3324 (64.2)	167 (72.0)	270 (71.4)	83 (78.3)	314 (62.7)	120 (64.5)	219 (63.5)	30 (58.8)	308 (68.6)	167 (68.4)	303 (60.7)	159 (64.9)	239 (80.5)	130 (61.9)	43 (74.1)	139 (75.5)	18 (46.2)	135 (74.6)	480 (49.2)
Smoking status - Never smoked, n(%)	408 (7.9)	10 (4.3)	31 (8.2)	5 (4.7)	25 (5.0)	61 (32.8)	24 (7.0)	4 (7.8)	9 (2.0)	11 (4.5)	40 (8.0)	31 (12.7)	9 (3.0)	31 (14.8)	12 (20.7)	6 (3.3)	0 (0.0)	12 (6.6)	87 (8.9)
Smoking status - Missing/NAs, n(%)	16 (0.3)	0 (0.0)	8 (2.1)	1 (0.9)	0 (0.0)	0 (0.0)	1 (0.3)	0 (0.0)	0 (0.0)	0 (0.0)	0 (0.0)	0 (0.0)	2 (0.7)	1 (0.5)	0 (0.0)	3 (1.6)	0 (0.0)	0 (0.0)	0 (0.0)
Smoking pack years, mean (SD)	39.9 (37.0)	73.1 (59.8)	38.1 (30.9)	68.6 (78.0)	39.0 (27.8)	27.9 (38.0)	36.8 (35.6)	30.7 (19.4)	53.3 (37.7)	34.8 (26.8)	41.4 (35.6)	38.3 (30.4)	52.6 (35.2)	30.3 (25.1)	50.1 (58.5)	30.4 (25.1)	31.1 (19.8)	28.9 (20.8)	30.7 (30.6)
FEV_1 measured in litres, mean (SD)	1.6 (0.7)	1.6 (0.7)	1.7 (0.8)	1.2 (0.6)	1.7 (0.8)	1.3 (0.6)	1.5 (0.7)	1.5 (0.7)	1.7 (0.7)	1.6 (0.7)	1.7 (0.7)	1.7 (0.8)	1.7 (0.7)	1.8 (0.7)	1.5 (0.7)	1.5 (0.7)	1.6 (0.6)	1.6 (0.7)	1.7 (0.7)
FEV_1 - Missing/NAs, n(%)	348 (6.7)	1 (0.4)	19 (5.0)	11 (10.4)	5 (1.0)	7 (3.8)	16 (4.6)	0 (0.0)	62 (13.8)	10 (4.1)	62 (12.4)	1 (0.4)	71 (23.9)	19 (9.0)	0 (0.0)	37 (20.1)	1 (2.6)	4 (2.2)	22 (2.3)
FEV_1 percent predicted, mean (SD)	60.6 (22.8)	57.7 (22.6)	62.3 (23.7)	46.5 (21.5)	65.0 (24.3)	58.7 (25.2)	52.5 (19.8)	54.0 (21.0)	60.3 (21.9)	57.1 (21.5)	60.3 (20.5)	62.6 (24.1)	68.5 (23.7)	66.9 (20.5)	66.4 (28.9)	50.1 (19.2)	54.1 (19.7)	59.2 (21.5)	63.1 (22.1)
FEV_1 percent predicted - Missing/NAs, n(%)	544 (10.5)	3 (1.3)	21 (5.6)	11 (10.4)	128 (25.5)	9 (4.8)	19 (5.5)	0 (0.0)	66 (14.7)	16 (6.6)	62 (12.4)	5 (2.0)	82 (27.6)	24 (11.4)	1 (1.7)	42 (22.8)	1 (2.6)	4 (2.2)	50 (5.1)
FVC measured in litres, mean (SD)	2.9 (1.0)	2.9 (0.9)	2.9 (1.0)	2.3 (0.9)	2.8 (1.0)	2.5 (0.9)	2.8 (0.9)	2.8 (1.0)	3.0 (0.9)	2.8 (0.9)	3.1 (0.9)	2.7 (1.0)	3.2 (0.8)	3.3 (0.9)	2.5 (0.9)	3.3 (1.1)	3.3 (0.8)	2.9 (1.1)	2.7 (0.9)
FVC - Missing/NAs, n(%)	361 (7.0)	1 (0.4)	20 (5.3)	11 (10.4)	6 (1.2)	7 (3.8)	30 (8.7)	0 (0.0)	62 (13.8)	6 (2.5)	64 (12.8)	1 (0.4)	71 (23.9)	19 (9.0)	0 (0.0)	37 (20.1)	1 (2.6)	4 (2.2)	21 (2.2)
FVC percent predicted, mean (SD)	82.1 (20.8)	81.6 (19.1)	82.6 (19.5)	73.3 (20.6)	83.1 (21.7)	83.3 (21.9)	75.2 (19.1)	76.1 (18.1)	82.0 (21.1)	77.3 (18.8)	87.5 (18.2)	77.2 (22.2)	97.6 (19.3)	98.9 (20.8)	82.6 (24.7)	86.8 (20.4)	85.1 (16.2)	84.1 (19.7)	76.8 (18.7)
FVC percent predicted - Missing/NAs, n(%)	557 (10.8)	3 (1.3)	22 (5.8)	11 (10.4)	129 (25.7)	9 (4.8)	33 (9.6)	0 (0.0)	66 (14.7)	12 (4.9)	64 (12.8)	5 (2.0)	82 (27.6)	24 (11.4)	1 (1.7)	42 (22.8)	1 (2.6)	4 (2.2)	49 (5.0)
BEC measured in 10^9/litre, geometric mean (SD)	0.16 (1.93)	0.13 (1.94)	0.16 (1.92)	NA (NA)	0.15 (1.97)	0.12 (1.92)	0.16 (1.76)	0.16 (1.77)	0.14 (1.92)	0.16 (2.03)	0.17 (1.93)	0.17 (1.76)	0.14 (2.01)	0.15 (1.72)	0.14 (2.01)	0.16 (2.07)	0.17 (1.76)	0.17 (1.91)	0.16 (1.91)

BEC - Missing/NAs, n(%)	2541 (49.0)	137 (59.1)	83 (22.0)	106 (100.0)	254 (50.7)	31 (16.7)	228 (66.1)	3 (5.9)	210 (46.8)	84 (34.4)	226 (45.3)	158 (64.5)	139 (46.8)	187 (89.0)	28 (48.3)	68 (37.0)	1 (2.6)	8 (4.4)	590 (60.5)
GOLD severity - GOLD 1, n(%)	949 (18.3)	38 (16.4)	86 (22.8)	8 (7.5)	103 (20.6)	38 (20.4)	28 (8.1)	8 (15.7)	74 (16.5)	36 (14.8)	71 (14.2)	60 (24.5)	70 (23.6)	57 (27.1)	21 (36.2)	11 (6.0)	5 (12.8)	30 (16.6)	205 (21.0)
GOLD severity - GOLD 2, n(%)	2048 (39.5)	101 (43.5)	146 (38.6)	27 (25.5)	163 (32.5)	60 (32.3)	141 (40.9)	18 (35.3)	175 (39.0)	100 (41.0)	227 (45.5)	103 (42.0)	94 (31.6)	85 (40.5)	17 (29.3)	52 (28.3)	15 (38.5)	83 (45.9)	441 (45.2)
GOLD severity - GOLD 3, n(%)	1206 (23.3)	54 (23.3)	96 (25.4)	35 (33.0)	73 (14.6)	63 (33.9)	111 (32.2)	21 (41.2)	104 (23.2)	65 (26.6)	108 (21.6)	54 (22.0)	43 (14.5)	38 (18.1)	10 (17.2)	55 (29.9)	16 (41.0)	46 (25.4)	214 (21.9)
GOLD severity - GOLD 4, n(%)	434 (8.4)	36 (15.5)	29 (7.7)	25 (23.6)	34 (6.8)	16 (8.6)	46 (13.3)	4 (7.8)	30 (6.7)	27 (11.1)	31 (6.2)	23 (9.4)	8 (2.7)	6 (2.9)	9 (15.5)	24 (13.0)	2 (5.1)	18 (9.9)	66 (6.8)
GOLD severity - Missing/NAs, n(%)	544 (10.5)	3 (1.3)	21 (5.6)	11 (10.4)	128 (25.5)	9 (4.8)	19 (5.5)	0 (0.0)	66 (14.7)	16 (6.6)	62 (12.4)	5 (2.0)	82 (27.6)	24 (11.4)	1 (1.7)	42 (22.8)	1 (2.6)	4 (2.2)	50 (5.1)
Taking ICS, n(%)	2926 (56.5)	142 (61.2)	245 (64.8)	88 (83.0)	301 (60.1)	123 (66.1)	160 (46.4)	29 (56.9)	178 (39.6)	120 (49.2)	262 (52.5)	115 (46.9)	137 (46.1)	124 (59.0)	39 (67.2)	149 (81.0)	24 (61.5)	112 (61.9)	578 (59.2)
Taking LABA, n(%)	3436 (66.3)	193 (83.2)	275 (72.8)	97 (91.5)	324 (64.7)	91 (48.9)	275 (79.7)	38 (74.5)	253 (56.3)	149 (61.1)	289 (57.9)	163 (66.5)	213 (71.7)	160 (76.2)	40 (69.0)	159 (86.4)	29 (74.4)	110 (60.8)	578 (59.2)
Taking LAMA, n(%)	3109 (60.0)	132 (56.9)	275 (72.8)	70 (66.0)	320 (63.9)	77 (41.4)	263 (76.2)	38 (74.5)	243 (54.1)	142 (58.2)	298 (59.7)	147 (60.0)	207 (69.7)	140 (66.7)	40 (69.0)	150 (81.5)	28 (71.8)	121 (66.9)	418 (42.8)
Cardiovascular comorbidity, n(%)	771 (14.9)	22 (9.5)	67 (17.7)	22 (20.8)	80 (16.0)	16 (8.6)	53 (15.4)	2 (3.9)	48 (10.7)	38 (15.6)	59 (11.8)	38 (15.5)	42 (14.1)	11 (5.2)	4 (6.9)	39 (21.2)	10 (25.6)	28 (15.5)	192 (19.7)
Domiciliary oxygen therapy, n(%)	151 (2.9)	5 (2.2)	6 (1.6)	4 (3.8)	15 (3.0)	31 (16.7)	2 (0.6)	0 (0.0)	5 (1.1)	10 (4.1)	6 (1.2)	23 (9.4)	2 (0.7)	2 (1.0)	0 (0.0)	7 (3.8)	1 (2.6)	1 (0.6)	31 (3.2)
Domiciliary oxygen therapy - Missing/NAs, n(%)	3352 (64.7)	140 (60.3)	193 (51.1)	60 (56.6)	357 (71.3)	97 (52.2)	291 (84.3)	29 (56.9)	270 (60.1)	162 (66.4)	190 (38.1)	132 (53.9)	258 (86.9)	167 (79.5)	41 (70.7)	102 (55.4)	18 (46.2)	135 (74.6)	710 (72.7)
mMRC - 0, n(%)	710 (13.7)	17 (7.3)	62 (16.4)	6 (5.7)	94 (18.8)	7 (3.8)	40 (11.6)	1 (2.0)	93 (20.7)	40 (16.4)	46 (9.2)	36 (14.7)	98 (33.0)	26 (12.4)	13 (22.4)	10 (5.4)	8 (20.5)	14 (7.7)	99 (10.1)
mMRC - 1, n(%)	1754 (33.9)	81 (34.9)	156 (41.3)	23 (21.7)	170 (33.9)	53 (28.5)	124 (35.9)	18 (35.3)	158 (35.2)	100 (41.0)	203 (40.7)	61 (24.9)	111 (37.4)	118 (56.2)	22 (37.9)	18 (9.8)	19 (48.7)	69 (38.1)	250 (25.6)
mMRC - 2, n(%)	1344 (25.9)	69 (29.7)	77 (20.4)	26 (24.5)	120 (24.0)	46 (24.7)	110 (31.9)	16 (31.4)	137 (30.5)	60 (24.6)	117 (23.4)	73 (29.8)	50 (16.8)	49 (23.3)	7 (12.1)	57 (31.0)	6 (15.4)	70 (38.7)	254 (26.0)
mMRC - 3, n(%)	939 (18.1)	48 (20.7)	64 (16.9)	27 (25.5)	78 (15.6)	48 (25.8)	51 (14.8)	15 (29.4)	50 (11.1)	30 (12.3)	89 (17.8)	58 (23.7)	24 (8.1)	13 (6.2)	8 (13.8)	31 (16.8)	5 (12.8)	21 (11.6)	279 (28.6)
mMRC - 4, n(%)	280 (5.4)	17 (7.3)	13 (3.4)	22 (20.8)	33 (6.6)	32 (17.2)	19 (5.5)	1 (2.0)	9 (2.0)	5 (2.0)	38 (7.6)	17 (6.9)	4 (1.3)	1 (0.5)	8 (13.8)	5 (2.7)	1 (2.6)	7 (3.9)	48 (4.9)
mMRC - Missing/NAs, n(%)	154 (3.0)	0 (0.0)	6 (1.6)	2 (1.9)	6 (1.2)	0 (0.0)	1 (0.3)	0 (0.0)	2 (0.4)	9 (3.7)	6 (1.2)	0 (0.0)	10 (3.4)	3 (1.4)	0 (0.0)	63 (34.2)	0 (0.0)	0 (0.0)	46 (4.7)
Rate of mod-to-severe exac at baseline, mean (SD)	0.55 (1.02)	0.56 (0.86)	0.74 (1.08)	0.60 (0.89)	0.42 (0.80)	0.60 (0.93)	0.21 (0.65)	0.92 (1.78)	0.60 (0.98)	0.50 (0.86)	1.28 (1.63)	0.53 (0.70)	0.18 (0.57)	0.30 (0.87)	0.28 (0.52)	0.94 (1.43)	0.82 (1.14)	0.37 (0.78)	0.38 (0.75)
Rate of severe exac at baseline, mean (SD)	0.18 (0.51)	0.24 (0.55)	0.21 (0.51)	0.45 (0.79)	0.11 (0.38)	0.52 (0.80)	0.04 (0.23)	0.08 (0.34)	0.30 (0.74)	0.18 (0.48)	0.14 (0.43)	0.24 (0.52)	0.09 (0.36)	0.08 (0.27)	0.16 (0.37)	0.24 (0.65)	0.31 (0.69)	0.12 (0.38)	0.17 (0.46)
Rate of mod-to-severe exac during Year 1, mean (SD)	0.44 (1.00)	0.33 (0.69)	0.48 (1.08)	0.34 (0.67)	0.34 (0.76)	0.40 (0.82)	0.23 (0.69)	0.59 (1.08)	0.59 (1.20)	0.34 (0.79)	0.96 (1.57)	0.63 (1.16)	0.20 (0.66)	0.21 (0.90)	0.28 (0.67)	0.88 (1.26)	0.74 (1.39)	0.38 (0.90)	0.27 (0.68)
Rate of severe exac during Year 1, mean (SD)	0.13 (0.49)	0.11 (0.37)	0.12 (0.47)	0.19 (0.48)	0.08 (0.34)	0.32 (0.77)	0.08 (0.32)	0.20 (0.49)	0.27 (0.84)	0.13 (0.53)	0.12 (0.42)	0.11 (0.42)	0.09 (0.34)	0.04 (0.20)	0.05 (0.22)	0.30 (0.70)	0.26 (0.64)	0.11 (0.45)	0.10 (0.37)
Average follow-up time in days, mean (SD)	356.8 (41.1)	358.0 (37.8)	348.9 (60.8)	345.5 (59.0)	356.0 (42.6)	357.1 (37.1)	357.2 (42.1)	361.9 (22.1)	362.0 (25.0)	351.4 (49.3)	354.8 (48.4)	363.9 (9.7)	360.9 (32.1)	353.0 (45.9)	360.4 (35.2)	360.4 (33.2)	365.0 (0.0)	357.4 (32.9)	357.4 (39.3)

*GOLD severity classification based on percent predicted forced expiratory volume in 1 second (FEV1P). GOLD 1: Mild (≥80%); GOLD 2: Moderate (50–79%); GOLD 3: Severe (30–49%); GOLD 4: Very Severe (<30%).
Abbreviations: BEC: Blood Eosinophil Count; BMI: Body Mass Index; FEV_1: Forced Expiratory Volume in 1 Second; FEV_1P: Forced Expiratory Volume in 1 Second expressed in Percent Predicted; FVC: Forced Vital Capacity; FVCP: Forced Vital Capacity expressed in Percent Predicted; GOLD: Global Initiative for Chronic Obstructive Lung Disease; ICS: Inhaled Corticosteroid; LABA: long-acting beta agonist; LAMA: long-acting muscarinic receptor antagonist; mMRC: modified Medical Research Council; SD: Standard Deviation.

Appendix Figure A2: Country-specific Kaplan-Meier survival curve

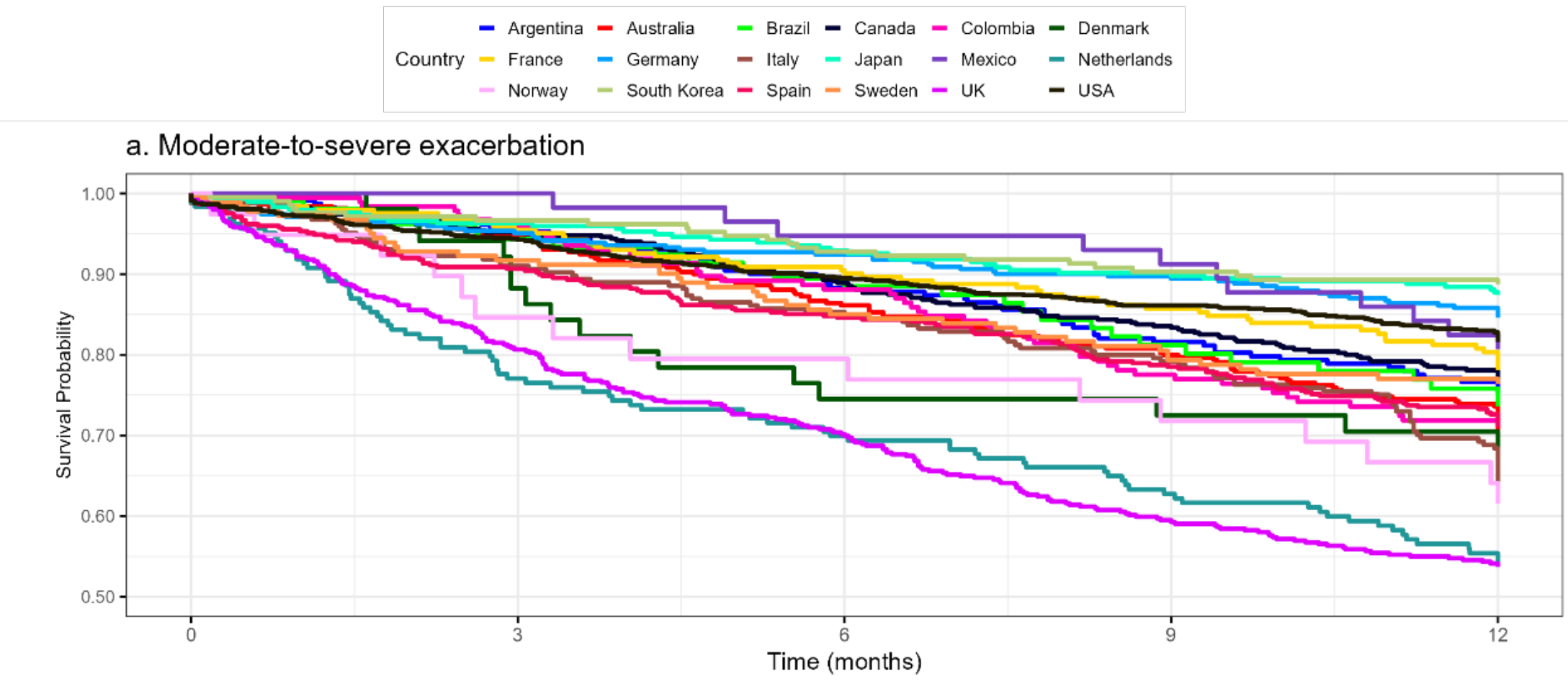

Number at risk

Country	0	3	6	9	12
Argentina	232	220	201	183	169
Australia	378	349	310	282	247
Brazil	106	100	91	76	69
Canada	501	475	436	403	364
Colombia	186	178	161	139	123
Denmark	51	45	38	36	35
France	244	232	215	194	177
Germany	345	326	311	299	283
Italy	245	223	209	193	164
Japan	297	284	272	263	254
Mexico	58	58	54	52	46
Netherlands	184	140	127	114	97
Norway	39	33	31	28	25
South Korea	210	202	191	180	168
Spain	449	406	377	347	318
Sweden	181	166	152	138	123
UK	499	397	338	283	245
USA	976	914	856	808	758

Time (months)

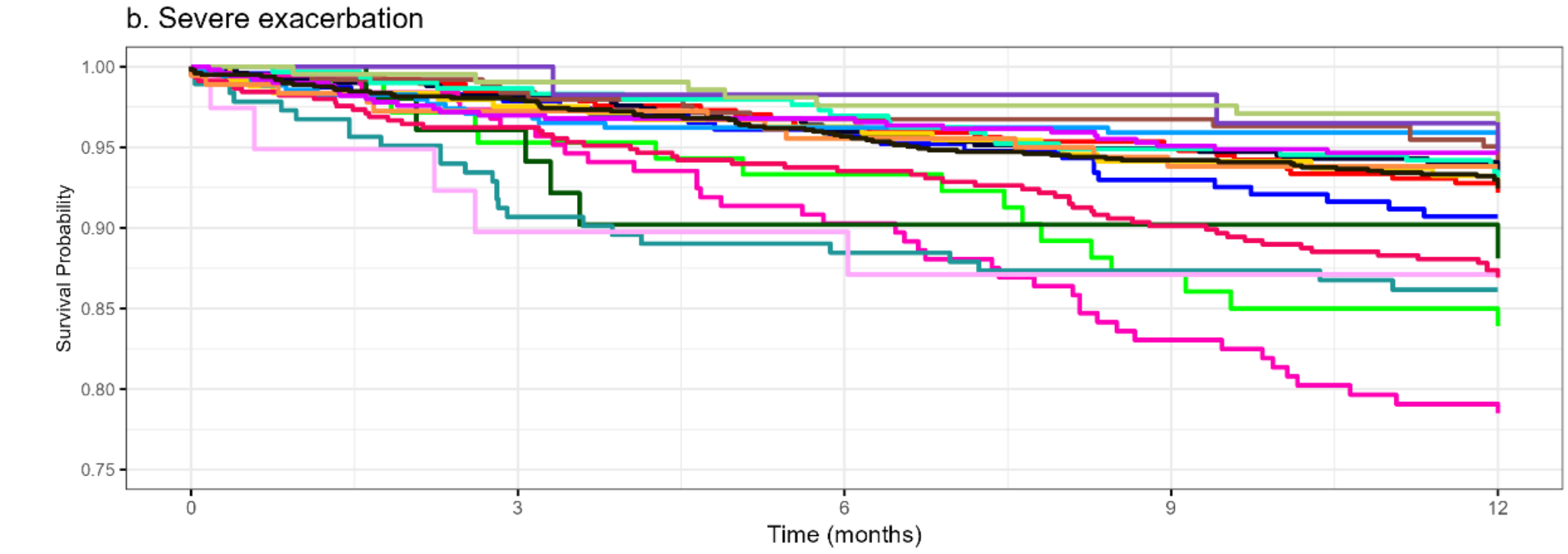

Number at risk

Country	0	3	6	9	12
Argentina	232	226	218	207	199
Australia	378	361	345	335	313
Brazil	106	101	95	82	78
Canada	501	489	470	457	439
Colombia	186	179	165	149	136
Denmark	51	49	45	44	43
France	244	237	229	213	203
Germany	345	332	322	317	313
Italy	245	241	237	233	223
Japan	297	290	282	275	269
Mexico	58	58	56	56	54
Netherlands	184	164	158	154	146
Norway	39	35	34	33	32
South Korea	210	207	201	194	183
Spain	449	431	416	396	379
Sweden	181	174	169	162	151
UK	499	476	464	448	422
USA	976	948	914	881	848

Time (months)

Appendix Figure A3: Country-specific rates of moderate and severe AECOPD

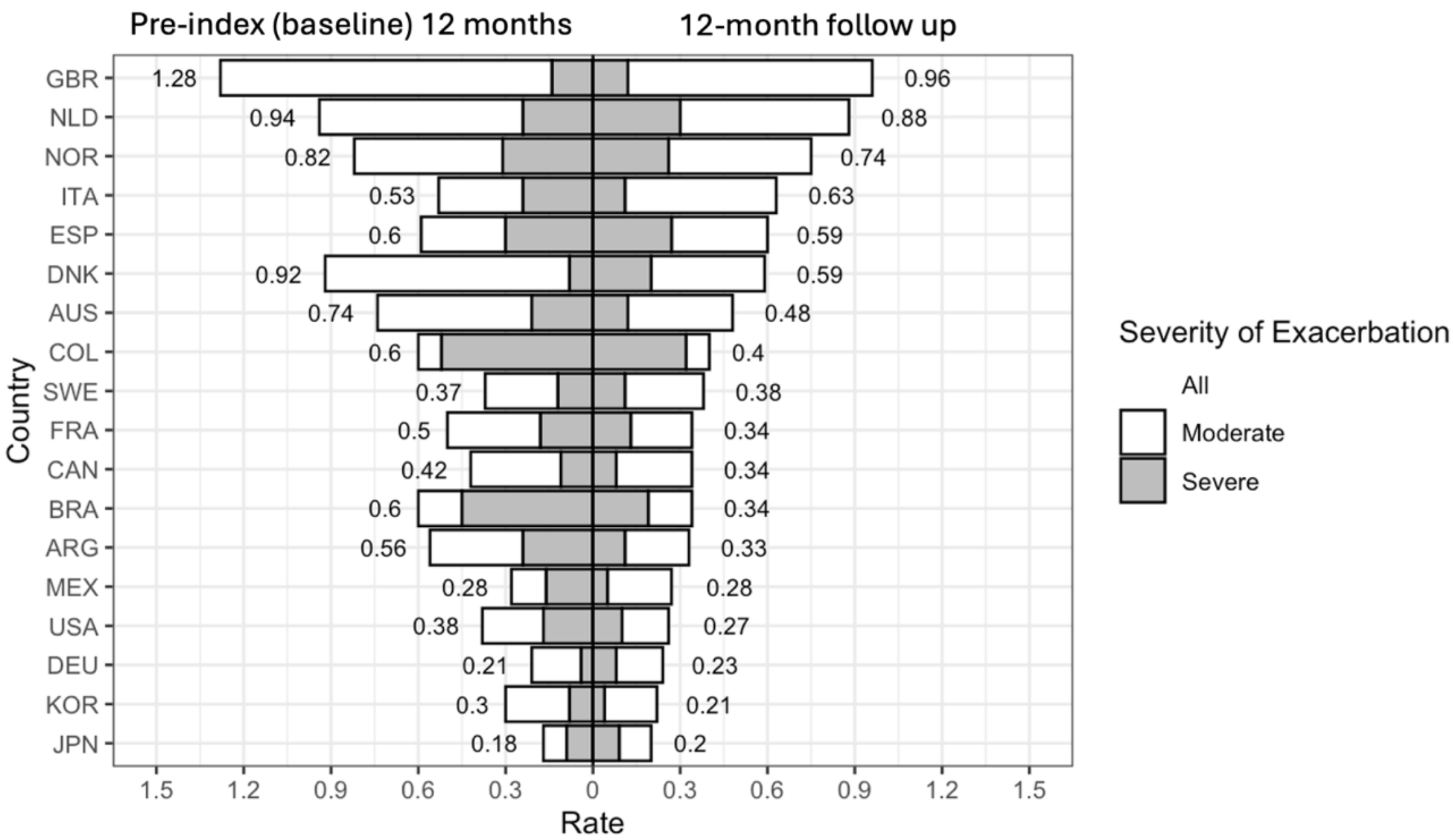

Appendix Figure A4: ROC curves of ACCEPT 2.0 and ACCEPT 3.0

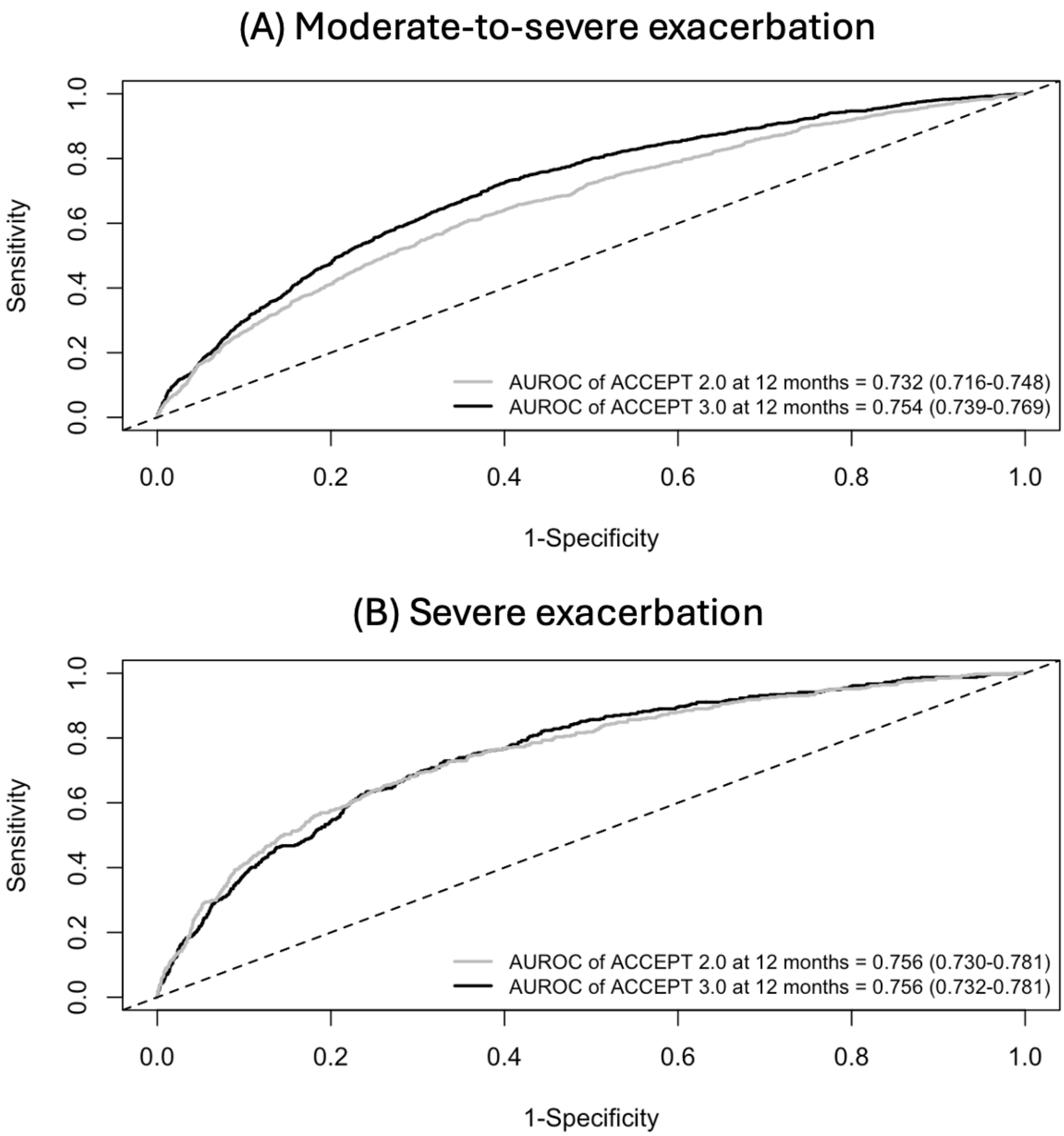

Appendix Figure A5: Country-specific calibration plots of ACCEPT 2.0 and ACCEPT 3.0

(A) Moderate-to-severe AECOPD: ACCEPT 2.0

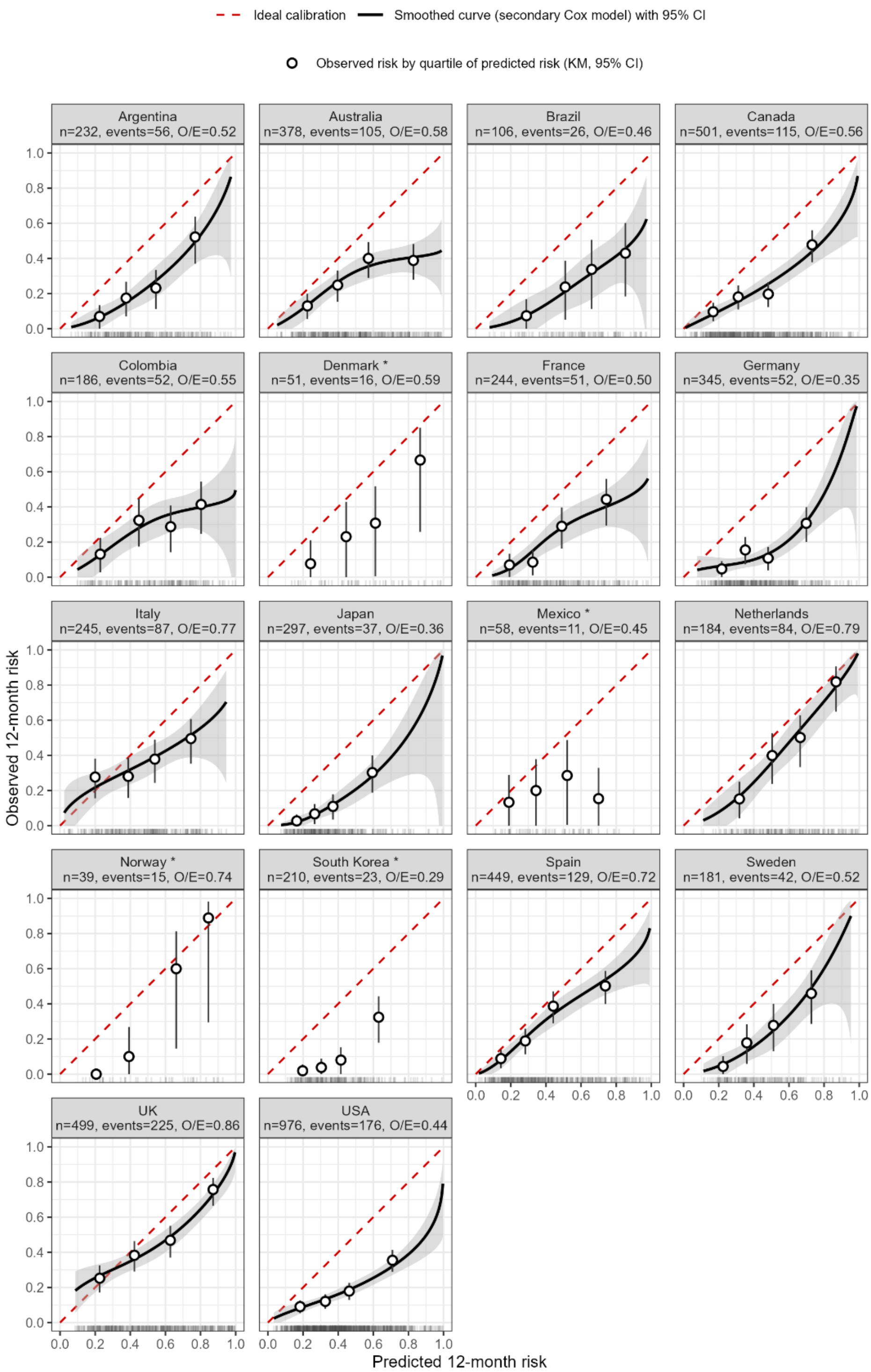

* Fewer than 25 events: smoothed curve not estimated; quartile points shown only.
Rug marks show individual predicted risks. O/E = observed (Kaplan-Meier) / mean predicted risk at 12 months.

(B) Moderate-to-severe AECOPD: ACCEPT 3.0

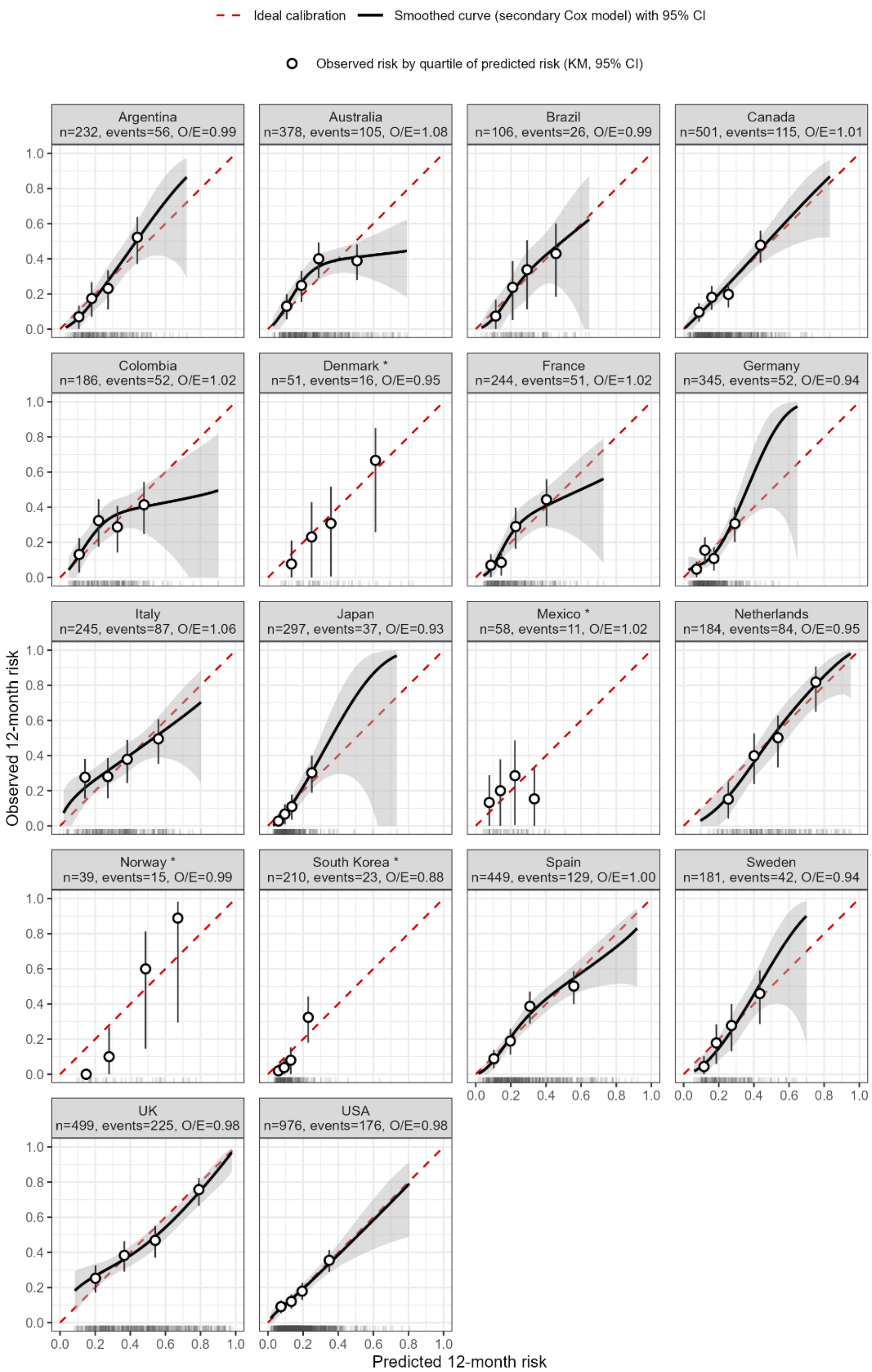

* Fewer than 25 events: smoothed curve not estimated; quartile points shown only.
Rug marks show individual predicted risks. O/E = observed (Kaplan-Meier) / mean predicted risk at 12 months.

(C) Severe AECOPD: ACCEPT 2.0

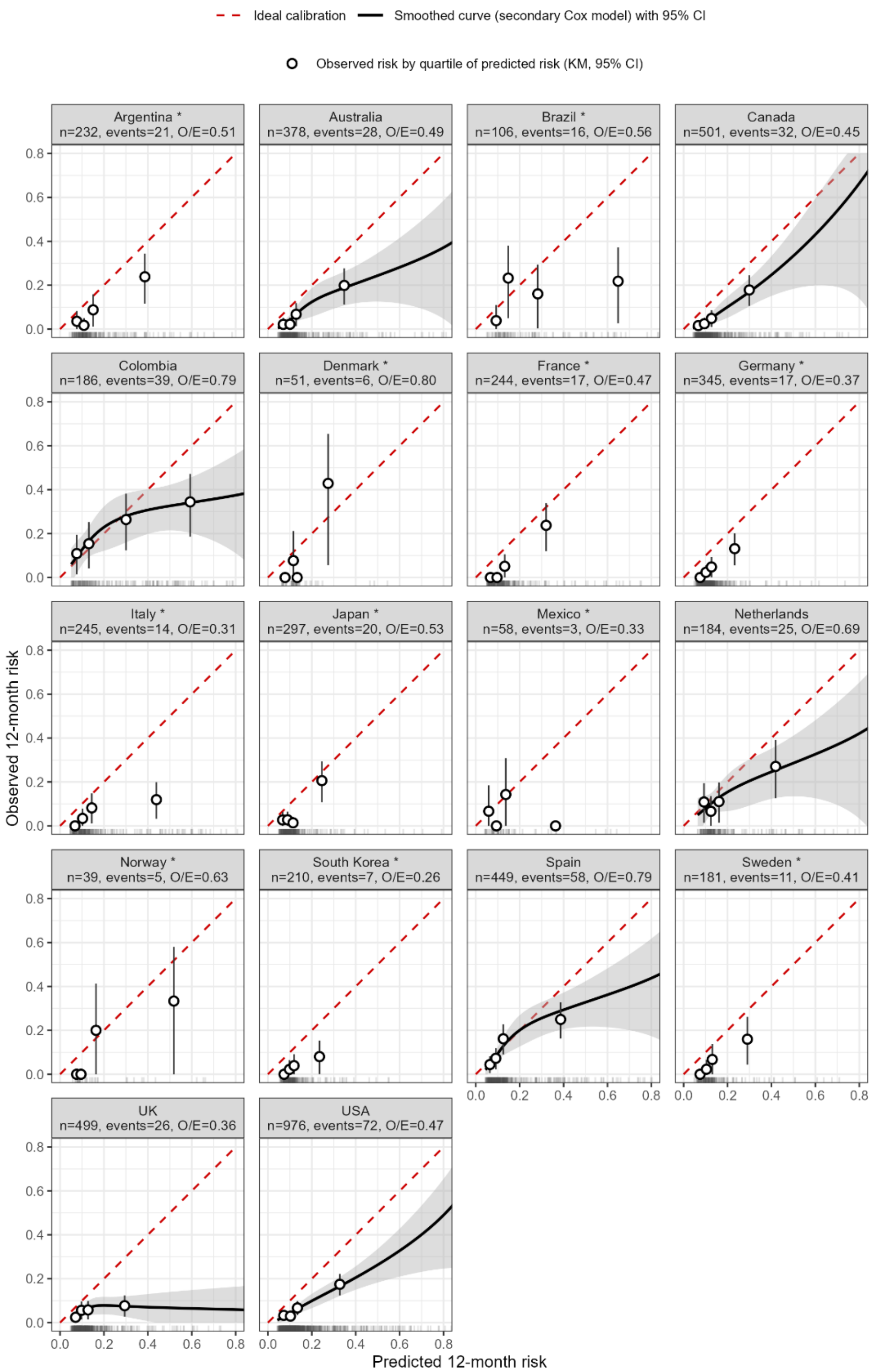

* Fewer than 25 events: smoothed curve not estimated; quartile points shown only.
Rug marks show individual predicted risks. O/E = observed (Kaplan-Meier) / mean predicted risk at 12 months.

(D) Severe AECOPD: ACCEPT 3.0

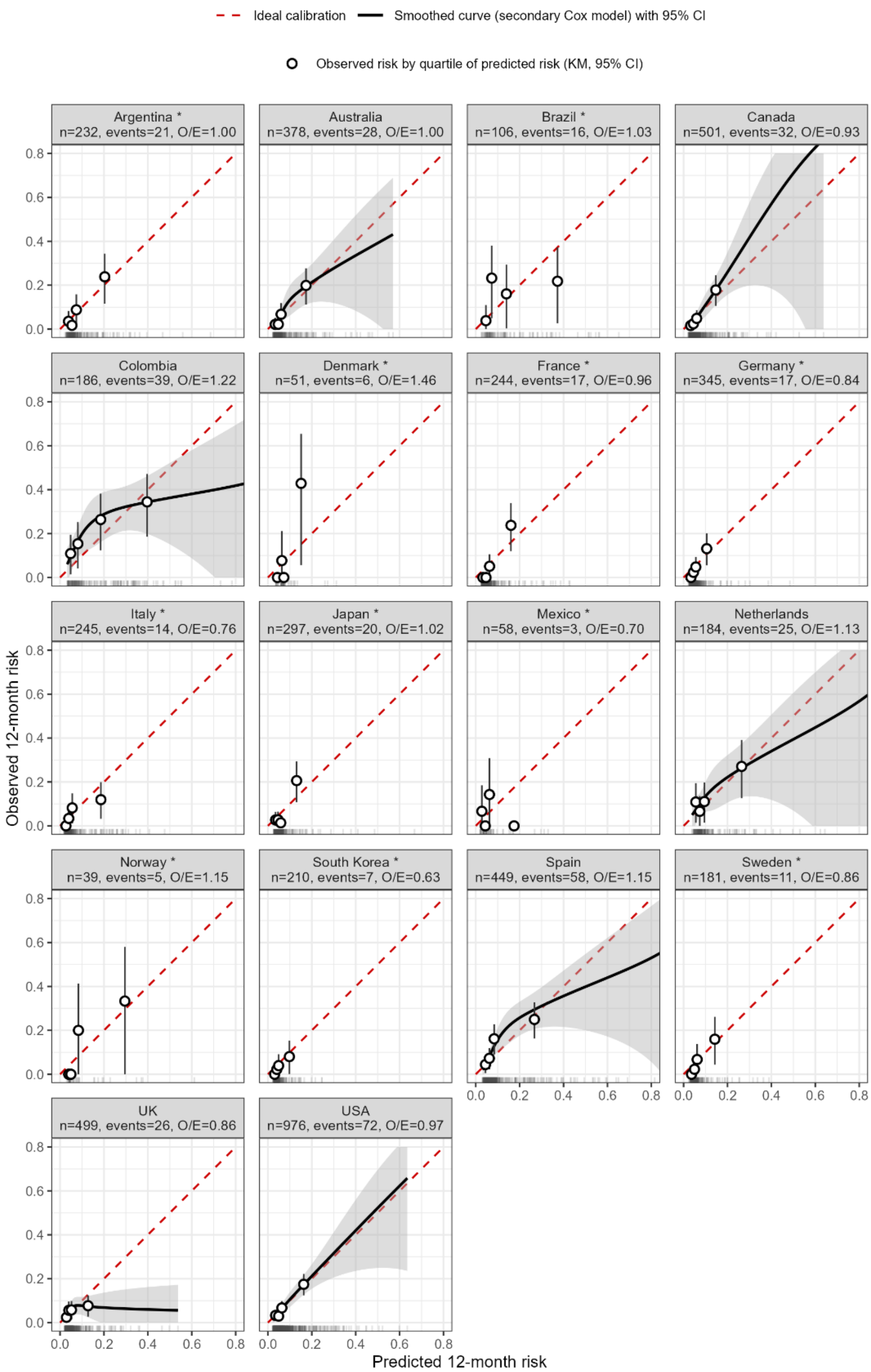

* Fewer than 25 events: smoothed curve not estimated; quartile points shown only.
Rug marks show individual predicted risks. O/E = observed (Kaplan-Meier) / mean predicted risk at 12 months.

Appendix Figure A6: Relationship between country-specific observed risk and intercept in ACCEPT 3.0

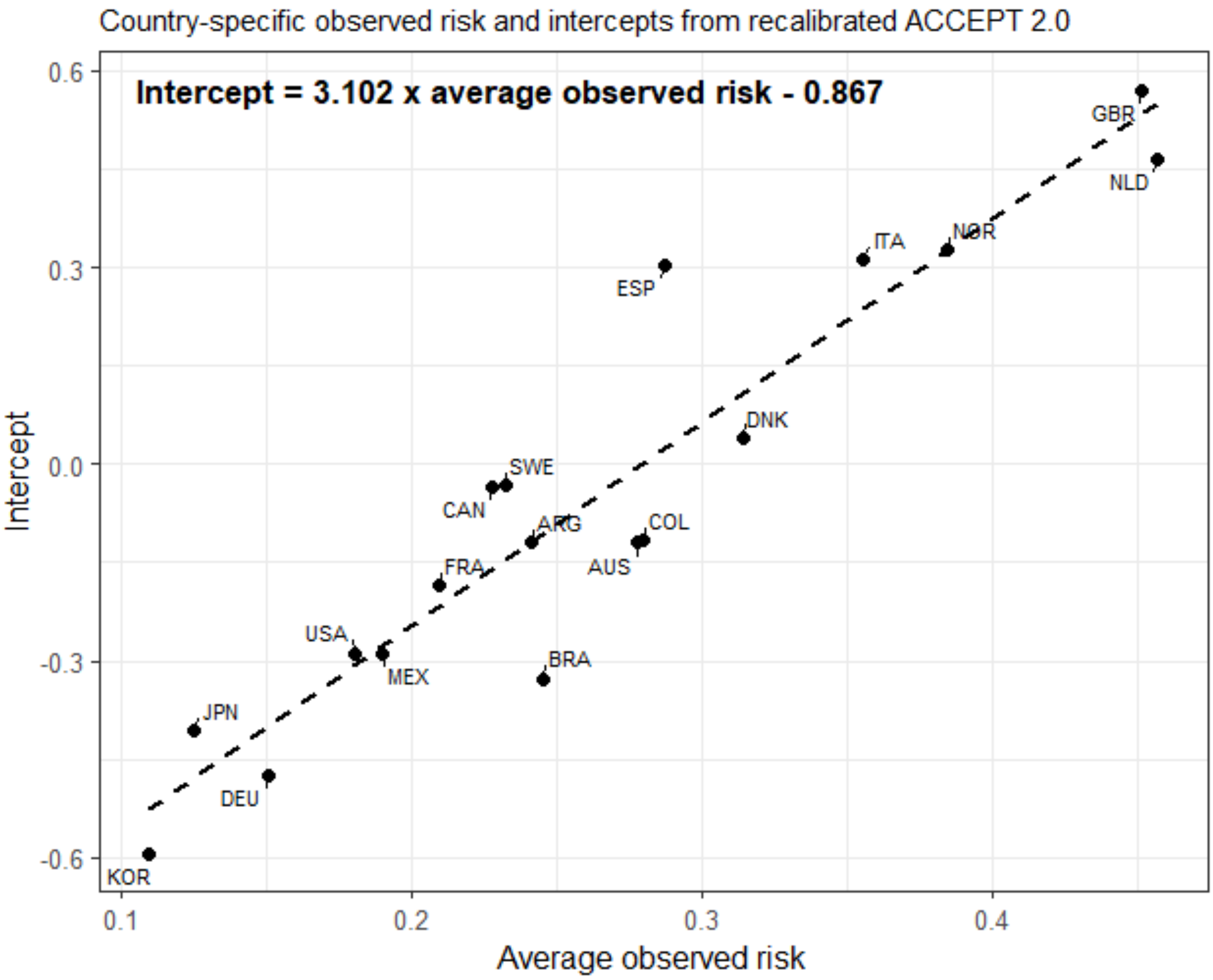

Note: Country-specific observed risks (x-axis) were estimated from the NOVELTY data, and the country intercepts (y-axis) were obtained from ***Table 2***. The relationship between country-specific observed risk and the corresponding intercept in ACCEPT 3.0 is approximately linear (p-value of slope <0.0001, adjusted R-squared = 0.877), allowing the updating of ACCEPT 2.0 for any setting where AECOPD risk is known.

Appendix Figure A7: Kaplan-Meier survival curve of 12-month moderate-to-severe and severe AECOPD in COPD patients with FEV_1/FVC < 0.7

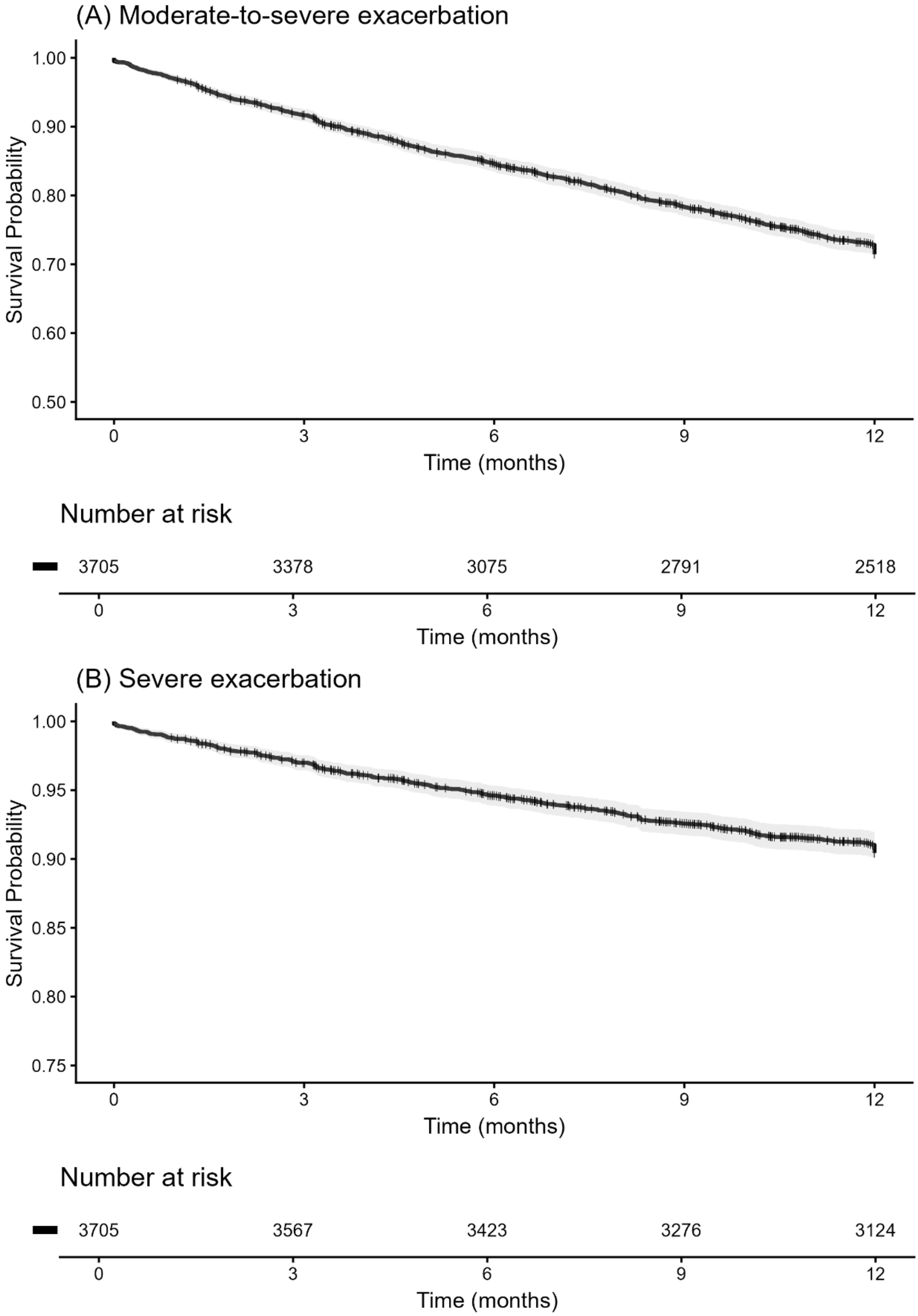

Appendix Figure A8: ROC curves of ACCEPT 2.0 and ACCEPT 3.0 in COPD patients with $FEV_1/FVC < 0.7$

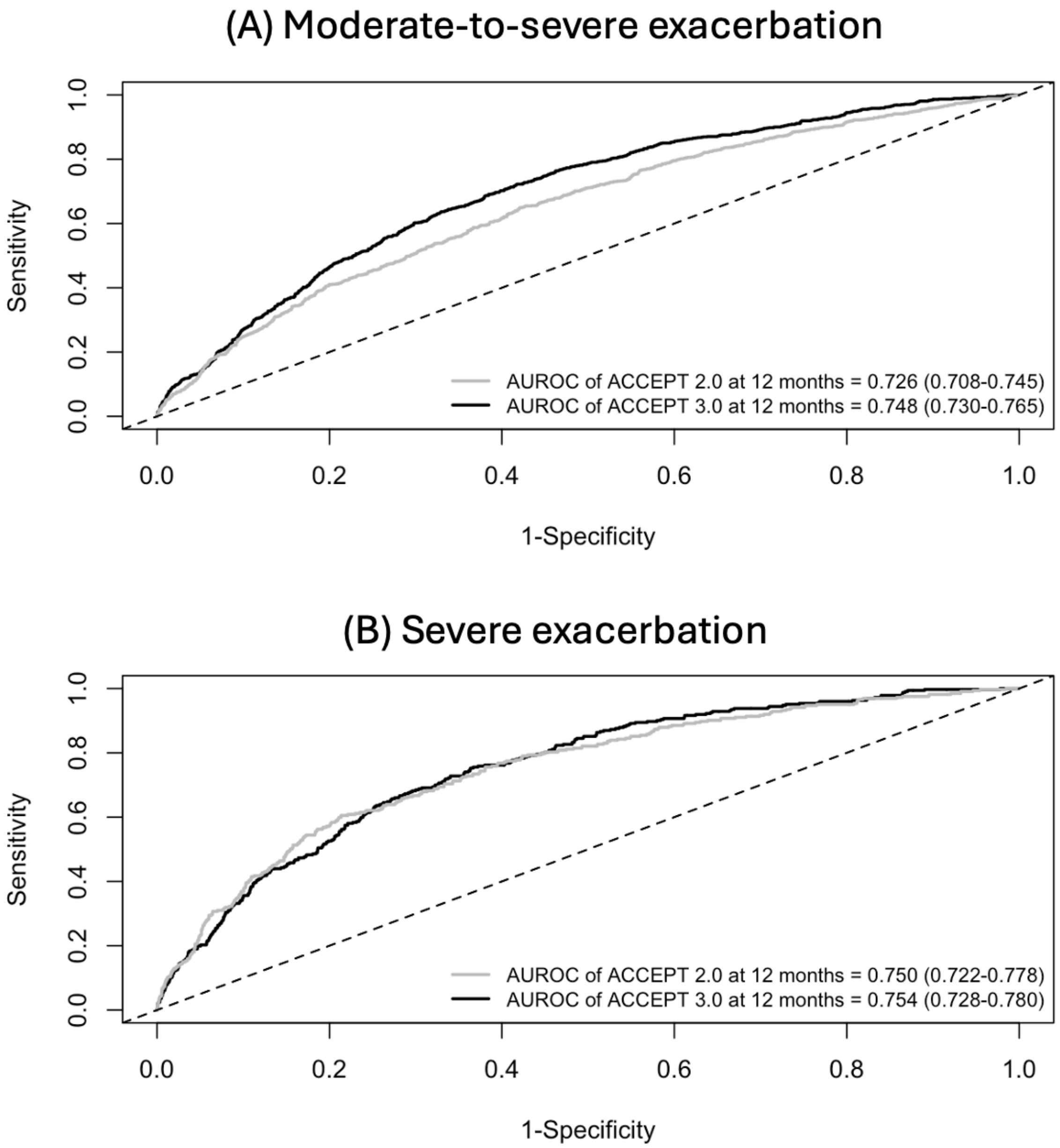

Appendix Figure A9: Calibration plots of ACCEPT 2.0 and ACCEPT 3.0 in COPD patients with FEV_1/FVC < 0.7

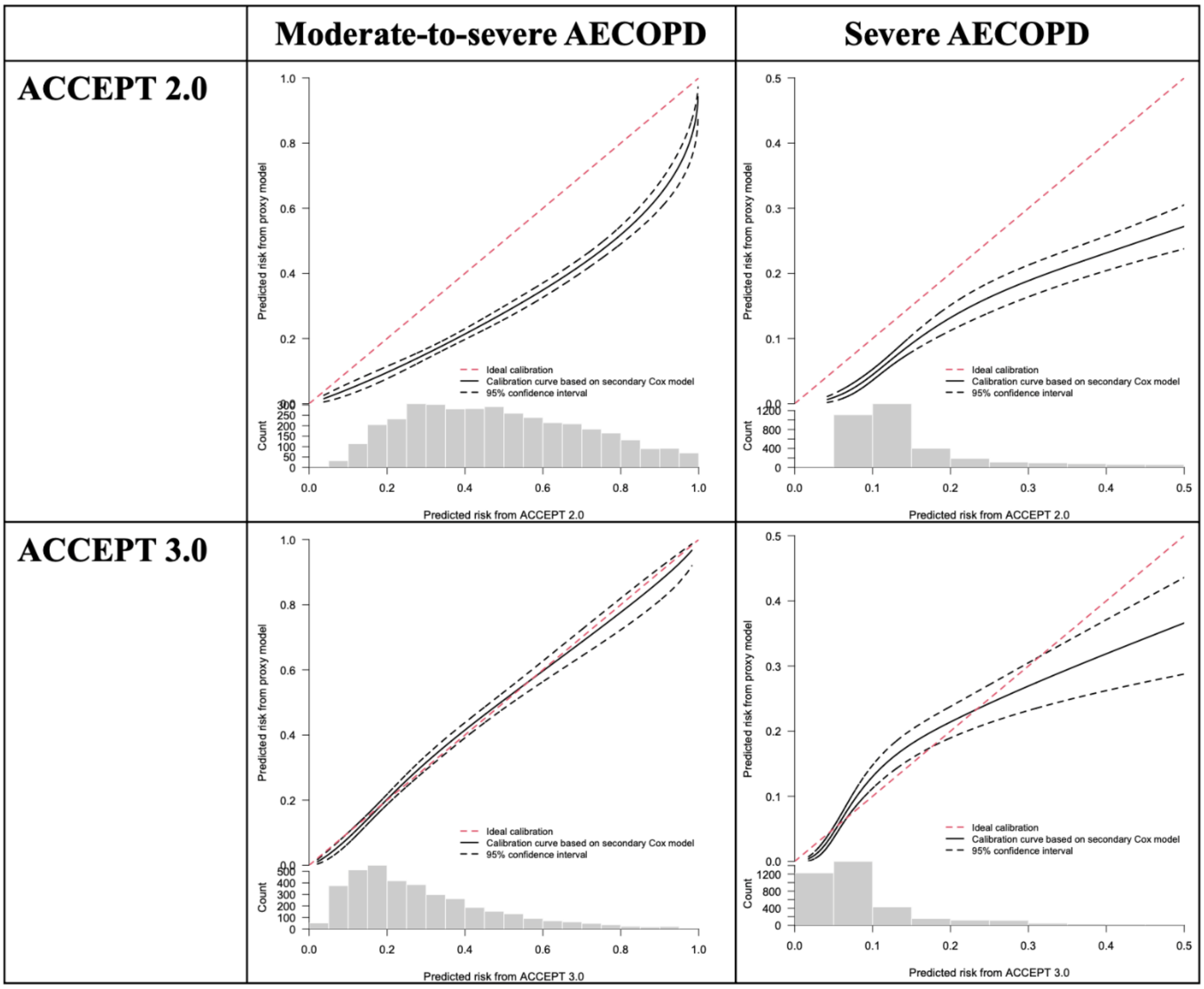

Appendix Figure A10: Decision curves of ACCEPT 2.0 and ACCEPT 3.0 in COPD patients with $FEV_1/FVC < 0.7$

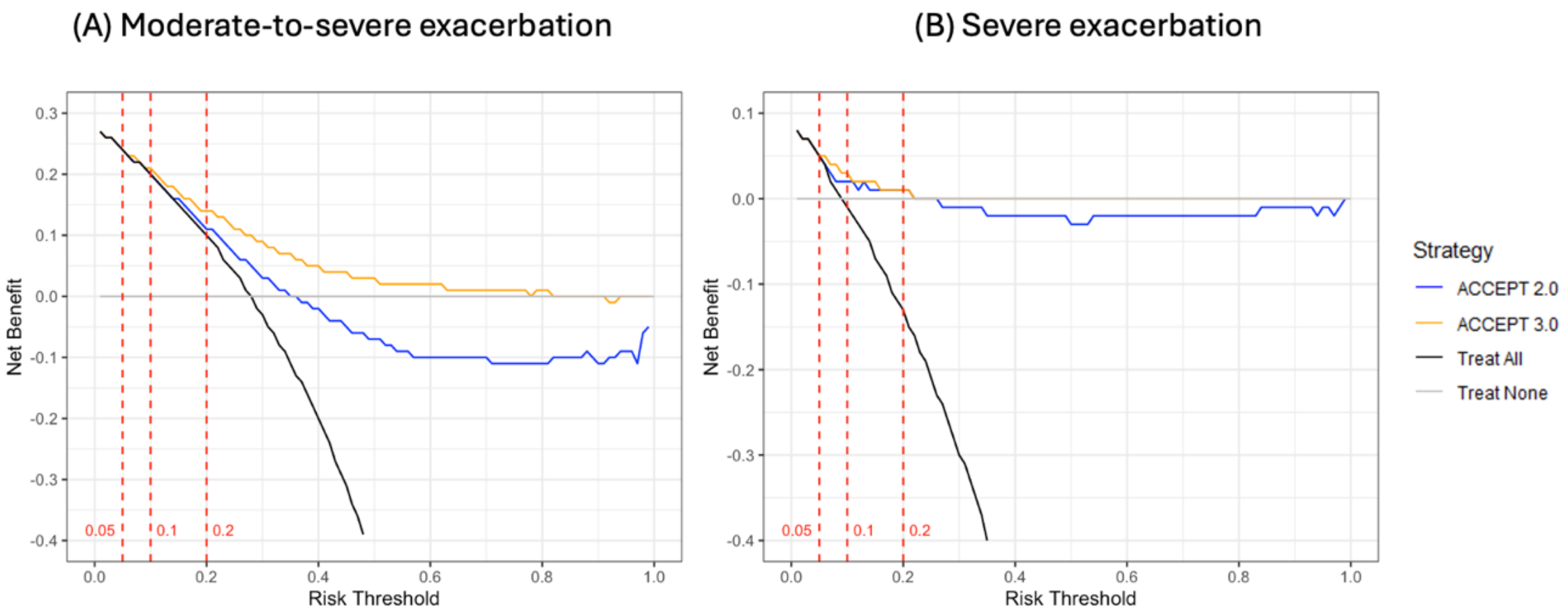

References

1. Sadatsafavi M, Sin DD, Zafari Z, Criner G, Connett JE, Lazarus S, et al. The Association Between Rate and Severity of Exacerbations in Chronic Obstructive Pulmonary Disease: An Application of a Joint Frailty-Logistic Model. Am J Epidemiol. 2016 Nov 1;184(9):681–9. doi:10.1093/aje/kww085 PubMed PMID: 27737842; PubMed Central PMCID: PMC5100830.

2. Albert RK, Connett J, Bailey WC, Casaburi R, Cooper JAD, Criner GJ, et al. Azithromycin for prevention of exacerbations of COPD. N Engl J Med. 2011 Aug 25;365(8):689–98. doi:10.1056/NEJMoa1104623 PubMed PMID: 21864166; PubMed Central PMCID: PMC3220999.

3. Criner GJ, Connett JE, Aaron SD, Albert RK, Bailey WC, Casaburi R, et al. Simvastatin for the prevention of exacerbations in moderate-to-severe COPD. N Engl J Med. 2014 Jun 5;370(23):2201–10. doi:10.1056/NEJMoa1403086 PubMed PMID: 24836125; PubMed Central PMCID: PMC4375247.

4. Aaron SD, Vandemheen KL, Fergusson D, Maltais F, Bourbeau J, Goldstein R, et al. Tiotropium in combination with placebo, salmeterol, or fluticasone-salmeterol for treatment of chronic obstructive pulmonary disease: a randomized trial. Ann Intern Med. 2007 Apr 17;146(8):545–55. doi:10.7326/0003-4819-146-8-200704170-00152 PubMed PMID: 17310045.

5. Agusti A, Calverley PM, Celli B, Coxson HO, Edwards LD, Lomas DA, et al. Characterisation of COPD heterogeneity in the ECLIPSE cohort. Respir Res. 2010 Sep 10;11(1):122. doi:10.1186/1465-9921-11-122

6. Yii A, Xu X, Loh CH, Bahety P, Navarro Rojas AA, Milea D, et al. Year-to-year trajectories of hospital utilisation rates among patients with COPD: a real-world, single-centre, retrospective cohort study. BMJ Open. 2023 Sep 1;13(9):e072571. doi:10.1136/bmjopen-2023-072571 PubMed PMID: 37657841; PubMed Central PMCID: PMC10476133.
